# The Prevalence and Control of *Bacillus* and Related Spore-Forming Bacteria in the Dairy Industry

**DOI:** 10.3389/fmicb.2015.01418

**Published:** 2015-12-21

**Authors:** Nidhi Gopal, Colin Hill, Paul R. Ross, Tom P. Beresford, Mark A. Fenelon, Paul D. Cotter

**Affiliations:** ^1^Teagasc Food Research CentreCork, Ireland; ^2^School of Microbiology, University College CorkCork, Ireland; ^3^APC Microbiome InstituteCork, Ireland; ^4^College of Science, Engineering and Food Science, University College CorkCork, Ireland

**Keywords:** dairy, spoilage, aerobic, spore-forming bacteria, biofilms

## Abstract

Milk produced in udder cells is sterile but due to its high nutrient content, it can be a good growth substrate for contaminating bacteria. The quality of milk is monitored via somatic cell counts and total bacterial counts, with prescribed regulatory limits to ensure quality and safety. Bacterial contaminants can cause disease, or spoilage of milk and its secondary products. Aerobic spore-forming bacteria, such as those from the genera *Sporosarcina, Paenisporosarcina, Brevibacillus, Paenibacillus, Geobacillus* and *Bacillus*, are a particular concern in this regard as they are able to survive industrial pasteurization and form biofilms within pipes and stainless steel equipment. These single or multiple-species biofilms become a reservoir of spoilage microorganisms and a cycle of contamination can be initiated. Indeed, previous studies have highlighted that these microorganisms are highly prevalent in dead ends, corners, cracks, crevices, gaskets, valves and the joints of stainless steel equipment used in the dairy manufacturing plants. Hence, adequate monitoring and control measures are essential to prevent spoilage and ensure consumer safety. Common controlling approaches include specific cleaning-in-place processes, chemical and biological biocides and other novel methods. In this review, we highlight the problems caused by these microorganisms, and discuss issues relating to their prevalence, monitoring thereof and control with respect to the dairy industry.

## Current Affairs in the Dairy Industry

The dairy industry is a dynamic global business, which plays an important role in the sustainability of the economies of many countries. According to the International Dairy Federation (IDF), 748.7 million tons of raw milk was produced in 2011, of which cow’s milk accounted for 620.7 million tons. Taking into account that dairy production has steadily been growing since 2000 (International Dairy Federation [IDF], 2013), it is not surprising that the Food and Agriculture Organization of the United Nations (FAO) estimated global raw milk production to be worth 292 billion USD in 2011, while trade in dairy products (such as cream, butter, cheese, whey, buttermilk, skim and whole milk powders, casein, yogurt, lactose, and infant formula) represented 69 billion USD. Meanwhile, in the European Union, raw milk, and dairy products were valued at 53.1 billion EUR, which accounts for 14% of the total value of the EU agricultural output in 2011 (Marquer, 2013). In 2013, according to the European Commission milk statistics, the estimated average milk yield across the EU-28 was 6.411 ton/head, with Denmark, Sweden and Finland having the highest average milk yield per cow (Eurostat website, 2013 – http://ec.europa.eu/eurostat).

With the abolition of milk quotas in April 2015, it is expected that milk producers across the EU will be facing new and demanding economic challenges. The European Commission, in its ‘milk package’ provisions, has already anticipated that there will be a potential increase in milk production, especially across quota-bound countries such as Ireland, Germany, the Netherlands, Denmark, Austria, Poland, and France (European Commission, 2014). Consequently, with the favorable trade in milk and more prominent consumption of dairy products across households, it is anticipated that dairy ingredients will be generated on a much larger scale to meet these growing demands. However, to match up with the high-scale production, it is essential for processors to ensure that processing capacity meets the expected increased milk supply without comprising on quality and customer expectations. Therefore, quality issues affecting the dairy industry will need to be assessed and addressed to maintain product quality and safety. The continued control of *Bacillus* and related spore-forming bacteria will be key in this regard.

## Determining the Quality of Milk

Milk present in healthy udder cells is sterile (Tolle, 1980). However, it can subsequently become contaminated *via* different sources such as the external surfaces of animals or from other environments during milking, transport, storage, or processing. An amalgam of different factors, such as composition, udder health and hygiene, are assessed to determine the quality of raw milk (O’Brien et al., 2009).

Milk quality, good hygiene, and farm management practices are frequently monitored according to total bacterial count (TBC) testing of the product and relates to the total number of living bacteria per ml of milk. A failure to refrigerate appropriately (Blowey and Edmondson, 2010) can also lead to an increase in TBC. The legal TBC limit in farm raw milk is set at 100,000 cells/ml (based on a geometric average over 2 months with at least two tests per month) across the EU and the Americas, with most farms striving for a desirable and achievable count of around 10,000 cells/ml (derived from Hillerton and Berry, 2004). In the dairy industry, it can be assumed that any grade A unpasteurized milk that has a TBC of less than 100,000 cells/ml will be cleared of pathogenic and most non-pathogenic bacteria during pasteurization (Pantoja et al., 2012). Among the other microbiology-based tests that are carried out to assess the quality and safety of milk and resultant dairy products, are those designed to quantify thermoduric populations and specific pathogens, such as *B. cereus*. Whilst the monitoring and control of foodborne pathogens is traditionally performed using culture-dependent approaches and standard biochemical identification, the advent of rapid, highly specific and highly sensitive detection methods has resulted in time-efficient, labor-saving, and reliable approaches (Mandal et al., 2011; Chapela et al., 2015; Law et al., 2015). These rapid methods are divided into three specific categories – nucleic acid-based assays, immunological methods, and biosensors (Mandal et al., 2011). Thus, controlling the levels of *Bacillus* and related spore-forming bacteria is key.

## *Bacillus* and Related Spore-Forming Bacteria and the Dairy Industry

As milk is highly nutritious and has a near neutral pH and high water activity, it provides an ideal environment for the proliferation of microorganisms (Quigley et al., 2013a). The microbiota of raw milk is rather complex, with the microbial community differing in response to hygiene, seasonality, animal species, animal health, and a variety of other factors (Quigley et al., 2013b). Of the microorganisms that can enter into the milk chain on farms or through dairy processing lines, the spore-forming bacteria are a particular concern as they have the ability, when in the spore form, to withstand harsh environmental conditions (Logan and De Vos, 2009; Postollec et al., 2012). Spore-formers are a primary cause of concern for manufacturers of powdered dairy ingredients and can be sub-categorized as being thermophilic, mesophilic or psychrotolerant in nature, with thermophilic spore-formers being more prevalent in the end product. The US Dairy Export Council has set strict tolerances for mesophilic (<1000 cfu/g) and thermophilic spores (<500 cfu/g) in dairy powders and hence, understanding the factors that lead to their proliferation and survival within processing plants is necessary to control and reduce their presence (Watterson et al., 2014).

Unfortunately, spore-forming bacteria are ubiquitous bacteria commonly found in the soil as well as being natural colonizers of the gastrointestinal tract of insects and warm-blooded animals (Postollec et al., 2012). They are Gram-positive organisms encompassing more than 200 species that are capable of forming endospores, which make them resistant to extreme conditions such as pressure, extreme heat or cold, drought, starvation, biocides, and UV irradiation (Moeller et al., 2008). Spore-forming bacteria belong to the phylum Firmicutes, which can further be divided into five distinct classes: *Bacilli, Clostridia, Erysipelotrichia, Negativicutes*, and *Thermolithobacteria* (Galperin, 2013; Zhang and Lu, 2015). Even with constant evolution and re-classifications, the *Bacilli* and *Clostridia* remain the most dominant classes within the Firmicutes phylum, consisting of 16 and 21 families, respectively (Galperin, 2013) and are arguably the most important classes that are relevant to the dairy industry.

Anaerobic spore-forming bacteria of the *Clostridium* genus, especially *C. botulinum* and *C. perfrigens*, are thought to be the most lethal foodborne microorganisms due to the array of potent toxins and neurotoxins they produce. The prevalence and significance of *Clostridium* sp. in the dairy industry has recently been reviewed by Doyle et al. (2015) and will, therefore, not be the focus of further discussion in this review. Meanwhile, the aerobic spore-forming bacteria (*Bacillus* and related genera) greatly impact on quality, food safety, and the economy due to their spoilage-causing capabilities and to a lesser extent, disease-causing potential. In an attempt to give an insight into this broad area, the following genera will be briefly described in this review: *Bacillus, Geobacillus, Paenibacillus, Brevibacillus, Sporocarcina*, and *Paenisporosarcina*.

### The *Bacillus* Genus

The *Bacillus* genus, part of the Bacillaceae family, is probably the oldest and most diverse genus of bacteria. As per the review of Slepecky and Hemphill (2006), *Bacillus* sp. have a remarkable range of physiological characteristics that renders appropriate categorization and generalizations impossible. Although their main habitat has always been considered to be the soil, it is now being suggested that soil may simply act as a reservoir for *Bacillus* sp. (Hong et al., 2009). Indeed, it would seem that their expansive physiology has allowed these species to colonize almost all natural environments including soil, air, lake sediments, water, and fodder as well as extreme environments such as thermal acid water, salt marshes, hot springs, sub-Antarctic soil, and diseased bee larvae (Claus and Berkeley, 1986).

Consisting of over 280 validly published species to date (LPSN www.bacterio.net), most members of this genus are considered non-pathogenic with only *B. anthracis* and *B. cereus* as outright exceptions. Nonetheless, an in-depth analysis into the *Bacillus* genus, by De Jonghe et al. (2010), has demonstrated that although species other than *B. cereus* have not been implicated in food poisoning cases, cellular assays have confirmed the production, and functionality of heat-labile toxins by *Bacillus circulans, Bacillus lentus, B. subtilis* (Beattie and Williams, 1999), *Bacillus licheniformis* (Beattie and Williams, 1999; Lindsay et al., 2000), *Bacillus pumilus* (Lindsay et al., 2000), and *Bacillus amyloliquefaciens* (Phelps and McKillip, 2002) and cereulide-resembling toxins by *B. licheniformis* (Beattie and Williams, 1999; Salkinoja-Salonen et al., 1999; Mikkola et al., 2000; From et al., 2005; Taylor et al., 2005), *B. pumilus* (Suominen et al., 2001; From et al., 2005, 2007), *B. amyloliquefaciens* (Mikkola et al., 2004, 2007), *Bacillus mojavensis* (From et al., 2007), *B. subtilis* (Beattie and Williams, 1999; From et al., 2005), *Bacillus simplex, Bacillus firmus, Bacillus megaterium* (Taylor et al., 2005), *B. circulans* and *B. lentus* (Beattie and Williams, 1999).

#### Bacillus cereus

Most relevant to this review, therefore, is the pathogenic soil-dweller *B. cereus* that is commonly encountered in raw milk and subsequent dairy products. The various strains of this species produce several potentially pathogenic substances such as hemolysins, phospholipases C, the emesis-causing toxin (Cereulide), enterotoxins, metalloproteinases, collagenases, and beta-lactamases (Turnbull et al., 2002). *B. cereus* has been linked to foodborne emetic and diarrheal syndromes and has been a known causative agent of food poisoning for more than 40 years (Ghelardi et al., 2002).

The emetic syndrome is caused by cereulide, which is synthesized by a non-ribosomal peptide synthetase that is encoded by the *ces* genes (Ehling-Schulz et al., 2005; Rajkovic et al., 2008). Cereulide is a small, heat-stable circular dodecadepsipeptide that has been shown to be toxic to mitochondria by acting as a potassium ionophore (Agata et al., 1995; Shinagawa et al., 1995; Mikkola et al., 1999), can cause cellular damage in animal models (Ehling-Schulz et al., 2005) and have an immunomodulating effect that can inhibit human natural killer cells (Paananen et al., 2002). The emetic toxin is produced by cells growing in the contaminated food (Kramer and Gilbert, 1989), and causes nausea and vomiting within 1–5 h of ingestion (Gilbert and Kramer, 1984; Schoeni, 2005). Although the exact number of *B. cereus* cells needed to produce adequate toxin to cause disease has not been determined, levels of 10^3^–10^10^ CFU g^-1^ have been detected in foods, which have caused outbreaks (Gilbert and Kramer, 1986).

The diarrheal syndrome, on the other hand, is caused by, at least, three known heat-labile enterotoxins: hemolysin BL (Hbl; Beecher et al., 1995), non- hemolytic enterotoxin (Nhe; Granum et al., 1999), and cytotoxin K (CytK; Lund et al., 2000). Hbl and Nhe each consists of three individual protein subunits while CytK belongs to the family of β-barrel pore-forming toxins (Stenfors Arnesen et al., 2008). Vegetative cells that are ingested as viable cells or spores produce protein enterotoxins in the small intestine causing the diarrheal syndrome, a mechanism sometimes termed a toxicoinfection (Granum et al., 1993; Andersson et al., 1998; Clavel et al., 2004). The main symptoms of *B.cereus*-related diarrhea include abdominal cramps and watery diarrhea 8–16 h after ingestion (Ehling-Schulz et al., 2005). The infective dose of the diarrheal disease has been described as ranging between 10^5^ and 10^8^ CFU g^-1^ of food although lower and much higher counts of viable cells or spores have also been reported (Gilbert and Kramer, 1986).

*Bacillus cereus* strains are able to survive industrial pasteurization processes due to the production of spores while psychrotrophic strains are able to survive refrigeration temperatures, and can affect the shelf-life of pasteurized milk and cream (Griffiths, 1992). Studies into the prevalence of aerobic spore-formers in raw milk have demonstrated that aerobic spore counts fluctuate with seasonal changes, with higher counts in the summer when cows are kept grazing outdoors as compared to the winter when cows are kept indoors (Christiansson et al., 1999). *B. cereus* is potentially the only food-poisoning causing pathogen in the *Bacillus* genus, but, several other species have been recognized as being the cause of bacterial spoilage of milk and milk products. Irrespective of seasonal, regional and methodological fluctuations, the microbiota of raw milk shows a few general trends – psychrotolerant strains of *B. cereus* are the predominant species in raw milk in the summer period (Scheldeman et al., 2006); *B. licheniformis, B. pumilus*, and *B. subtilis* represent the principal mesophilic spore-forming species (Phillips and Griffiths, 1986; Sutherland and Murdoch, 1994; Tatzel et al., 1994; Lukášová et al., 2001) while thermotolerant strains of *B. licheniformis* are the most prevalent thermophilic or thermotolerant species (Phillips and Griffiths, 1986).

#### Bacillus licheniformis

*Bacillus licheniformis*, alongside *B.cereus*, has been shown to be one of the most prevalent *Bacillus* species encountered in raw milk and along the dairy processing continuum (Kalogridou-Vassiliadou, 1992; Scheldeman et al., 2006). Although, *B. licheniformis* is not categorized as a human pathogen, the spores of the bacterium are known to cause spoilage of milk and dairy products, raise specification compliance problems and have adverse effects on milk organoleptic and functional properties (Crielly et al., 1994; Dhakal et al., 2014). Previous studies have demonstrated that *B. licheniformis* strains encountered in dairy foods show extensive genetic heterogeneity, with the high prevalence in the dairy industry attributed to contamination from external sources such as soil and silage as well as sources intrinsic to dairy processing factories (Rückert et al., 2004; Scheldeman et al., 2005; Dhakal et al., 2013). *B. licheniformis* is often associated with milk powders that have a low spore count due to its inability to form biofilms and is also known to be among spore-formers capable of producing spoilage enzymes (De Jonghe et al., 2010; Reginensi et al., 2011).

*Bacillus licheniformis* was the second most common thermophilic spore-former detected in a survey of 28 milk powder samples sourced from 18 different countries, with a total occurrence of 39.2% (Rückert et al., 2004). The same study highlighted the presence of a specific *B. licheniformis* strain in 27 out of the 28 milk powder samples, indicating its ubiquity as a common soil isolate (Rückert et al., 2004). Studies into the seasonal effects of the bacterial flora of farms in the Midwest, USA (bulk tank milk, environment, and corn silage) identified that *B. licheniformis* was the predominant species in summer (49.4%) as well as in winter (62%), albeit having lower numbers in summer (Buehner et al., 2014). In line with other studies, infant milk formula (IMF) and whole milk powders sampled from diverse manufacturers in China identified *B. licheniformis* as the most prevalent thermophilic bacilli in Chinese milk powders, with an occurrence of 36.8% out of 801 isolates (Yuan et al., 2012). The high prevalence of *B. licheniformis* in milk samples is potentially attributed to its widespread distribution in the environment and across the dairy farms, e.g., feed concentrate, fecal matter, soiled udders and teats, raw milk as well as from factory-derived contamination (Vaerewijck et al., 2001; Rückert et al., 2004; Scheldeman et al., 2005; Reginensi et al., 2011).

#### Bacillus thuringiensis

*Bacillus thuringiensis* forms part of the *B. cereus* group of bacteria (*B. cereus* sensu lato) and is known to be biochemically identical to *B. cereus* sensu stricto. The bacteria is known to produce similar enterotoxins as *B. cereus* sensu stricto, with some commercially used strains known to have genes for Hbl, Nhe, and CytK (Kim et al., 2014). However, due to the fact that standard methods for detection and enumeration (ISO7932 and ISO21871) in food and clinical settings do not differentiate between the two species, the occurence of outbreaks due to *B. thuringiensis* is often underestimated (Anonymous, 1997; European Food Safety Authority, 2005; Kim et al., 2014). Despite its known pathogenicity, *B. thuringiensis* has only been implicated in one confirmed case of food poisoning even though it has previously been isolated from a variety of food products including milk, farm bulk tank and creamery silo milk indicating the potential attribution of outbreaks to *B. cereus* sensu stricto rather than the actual *B. thuringiensis* (Phillips and Griffiths, 1986; Jackson et al., 1995; Damgaard et al., 1996). For years, commercial preparations of *B. thuringiensis* have been used as biological insecticides, since it is an entomopathogen capable of producing several insecticidal proteins (Lemes et al., 2015). Hence, spraying of agricultural crops with *B. thuringiensis* preparations to protect against insect infestations is common, raising the question of the prevalence of enterotoxins that are pathogenic to humans, should the bacterial preparations persist upon consumption.

#### Other *Bacillus* sp.

Other *Bacillus* species such as *B. weihenstephanensis, B. pumilus*, and *B. subtilis* often isolated from milk and farm environments have been reported to either produce putative cereulide, the emetic toxin produced by *B. cereus* or other enterotoxic substances that can be harmful to humans (Nieminen et al., 2007; Yoo et al., 2014). *B. weihenstephanensis* is a psychrotolerant bacterium known to cause spoilage but its pathogenicity has yet to be elucidated (Thorsen et al., 2006; Hoton et al., 2009; Stenfors Arnesen et al., 2011). *B. pumilus* is not known to commonly cause human infections but has previously been isolated from mastitic milk samples in Finland (Nieminen et al., 2007; Caamaño-Antelo et al., 2015). *B. subtilis*, on the other hand, although not traditionally categorized as a human pathogen, has been reported to be involved in food poisoning cases, e.g., in a 2005 outbreak in a kindergarten caused by milk powder, with vomiting being the most common symptom but is often accompanied by diarrhea (Pavic et al., 2005; Logan, 2012). However, it is believed that outbreaks caused by *B. weihenstephanensis, B. pumilus*, and *B. subtilis* are often attributed to *B. cereus*, unless further in-depth microbiological identifications are initiated (Logan, 2012). Another spore-forming bacterium encountered globally in milk products is the highly heat resistant spore-producing *B. sporothermodurans* (Pettersson et al., 1996). The occurrence of *B. sporothermodurans* has mostly been identified in indirectly UHT-processed milk and affected milk products ranged from whole, skimmed, evaporated, or reconstituted UHT milk to UHT cream, chocolate milk, and milk powders (Hammer et al., 1995; Klijn et al., 1997).

Because of their ubiquity in nature, the actual source of contamination by *Bacillus* sp. on dairy farms can seldom be determined exactly and is probably a cumulative process as raw milk is extracted, processed, transported, and stored. Most common contamination sources include soil, bedding material, silage, feces, rinse water from milking equipment, and feed (Christiansson et al., 1999; Magnusson et al., 2007). Once raw milk is contaminated by these spoilage species, the entire milk processing continuum is affected since some *Bacillus* sp. produce highly hydrophobic spores that can firmly adhere to inert materials used in food processing, e.g., stainless steel and polymers to form multicellular structures called biofilms (Wiencek et al., 1991; Hüsmark and Rönner, 1992). Once attached to manufacturing plants, these biofilms become a reservoir of spores, which act as the main contamination source of final dairy products indicating poor plant hygiene (Burgess et al., 2013). In the last section of this review, we address in greater detail the issue of biofilm formation.

### The *Geobacillus* Genus

The genus *Geobacillus* was established by Nazina et al. (2001) following reclassification of the thermophilic bacilli of genetic group 5 (group 5 is one of the five phylogenetically distinct clusters of Bacillus, based on 16S rDNA sequence alignment; Ash et al., 1991; Rainey et al., 1994; Xu and Côté, 2003; Kuisiene et al., 2004). Part of the Bacillaceae family, *Geobacillus* comprised of 19 species and four sub-species at the time of writing (LPSN www.bacterio.net). Various investigations have shown that *Geobacillus* strains are wide spread in the environment, having been isolated from high-temperature environments such as geothermal features or sub-surface layers of high-temperature oil fields as well as cooler environments, e.g., cool soil samples or hay compost (Sung et al., 2002; Banat et al., 2004; Marchant and Banat, 2010).

*Geobacillus* sp. are known to be among the most common contaminants of milk powders. As an obligate thermophile that grows at temperatures ranging between 48 and 60°C, *Geobacillus* is able to survive industrial pasteurization of raw milk and subsequently, spores adhere to surfaces and germinate to form biofilms, resulting in spoilage of milk products (Murphy et al., 1999; Seale et al., 2012). Although not pathogenic, members of this species and most importantly the aforementioned, *Geobacillus stearothermophilus*, is a proven problematic spore-forming bacterium encountered in whole and skim milk powders produced across the world (Rückert et al., 2004). An RAPD-based (random amplified polymorphic DNA) survey by Rückert et al. (2004) established that *G. stearothermophilus* accounted for 10.8% of all isolates (742) tested in milk powders originating from Poland, Germany, Netherlands, Great Britain, USA, Mexico, Chile, South Africa, Australia, and New Zealand. Meanwhile, a survey undertaken at a whole milk powder manufacturing plant in New Zealand reported that *Geobacillus* sp. were able to colonize all stages of the manufacturing process (evaporators, surface heaters, homogeniser, and vibrofluidiser) apart from the pre-heating process (Scott et al., 2007). The same study also highlighted that *Geobacillus* sp. was the predominant thermophilic spore type isolated from foulant samples collected from different sites of the processing stages after a manual shutdown, indicating a potential source of inoculum for *Geobacillus* sp. (Scott et al., 2007).

*Geobacillus* strains were also isolated from a biodiversity survey looking at spoilage and non-spoilage associated aerobic spore-formers in dairy processing and food products (Lücking et al., 2013). In this study, *Geobacillus* sp. represented 10% of the 467 investigated isolates with *G. stearothermophilus* (41 isolates) being the most prevalent *Geobacillus* strain followed by *G. pallidus* (five isolates) and *G. thermoleovorans* (two isolates; Lücking et al., 2013). Zhao et al. (2013), in their investigation into biofilms formed by thermophilic spore-formers, affirmed that the most potent biofilm-forming *Geobacillus* strains isolated from the dairy industry are *G. stearothermophilus* and *G. thermoglucosidans.* The study showed that *Geobacillus* species have greater ability to form biofilms at air-liquid interfaces rather than submerged surfaces, with the most stable spore-producing *G. thermoglucosidans* requiring proteolytic input from other spoilage organisms to thrive (Zhao et al., 2013). However, although *Geobacillus* sp. are common contaminants encountered in the dairy industry, not many reproducible genotypic assays to differentiate between different strains and their potential sources exist as routine testing relies on biochemical differences (Seale et al., 2012). Even though previous studies have used random amplification of polymorphic DNA PCR (RAPD_PCR) to genotype *Geobacillus* sp., poor reproducibility and inaccurate interpretation of banding patterns render the technique questionable (Seale et al., 2012). Other approaches such as genotyping of specific housekeeping genes have also been trialed but without much success, highlighting the need for research into novel genotypic assays (Durak et al., 2006).

### The *Paenibacillus* Genus

The *Paenibacillus* genus was coined by Ash et al. (1993) following a thorough investigation into the 16S rRNA gene sequences of 51 species of the *Bacillus* genus (Ash et al., 1991). This genus is now part of the Paenibacillaceae family and currently comprises of 154 named species (LPSN www.bacterio.net). The type species is *Paenibacillus polymyxa* (LPSN www.bacterio.net). Normal environments of *Paenibacillus* strains include soil, the rhizosphere of plants, water, diseased insect larvae, and food products (Daane et al., 2002; Ahn et al., 2014). As highlighted in a review by Heyndrickx and Scheldeman (2002), low numbers of *Paenibacillus* spores can be found in both raw and pasteurized milk. Although there had been no previous reports of these spores surviving industrial sterilization or UHT processing of milk, *Paenibacillus lactis* has been isolated directly from raw and UHT milk as well as from the dairy farm environment (Scheldeman et al., 2004). In that particular study, *P. lactis* was isolated in conjunction with *B. sporothermodurans*, a bacterium that produces endospores capable of resisting ultra-high temperature (UHT) and industrial sterilization processes (Heyndrickx et al., 2012). The simultaneous isolation of these two species indicates that *Paenibacillus* is also capable of resisting UHT and go on to have an impact on food safety and quality (Scheldeman et al., 2004). In the dairy farm environment, the most common sources of raw milk contamination with *Paenibacillus* strains are silage and feed concentrate for cattle (Vaerewijck et al., 2001; te Giffel et al., 2002).

Alongside *Bacillus* sp., *Paenibacillus* sp. has been classified as being an essential limiting factor in the shelf-life of high temperature short time (HTST) pasteurized fluid milk due to their ability to survive HTST pasteurization regimens as well as thrive at refrigeration temperatures (Fromm and Boor, 2004; Durak et al., 2006; Huck et al., 2007, 2008). A real-time PCR-based investigation into pasteurized fluid milk products by Ranieri et al. (2012) has indicated that, once post-pasteurization contamination is excluded, *Bacillus* sp. is more prevalent in raw milk and at early stages of shelf-life (<7 days). Meanwhile, *Paenibacillus* sp. is highly prevalent (over 95% of the bacterial population) late in shelf-life (>10 days; Ranieri and Boor, 2010) as they are psychrotolerant and grow extensively during refrigerated storage. However, due to the lack of appropriate molecular-based rapid detection assays for spoilage microorganisms such as *Paenibacillus* sp., it is only recently that *Paenibacillus* has been characterized as the predominant fluid milk spore-forming spoilage genus (Fromm and Boor, 2004; Huck et al., 2008; Ranieri and Boor, 2009; Ivy et al., 2012).

### Other Related Genera

The *Brevibacillus* genus was established by Shida et al. (1996) following genetic reclassification of species belonging to the *Bacillus brevis* group and now forms part of the Paenibacillaceae family (Sanders et al., 2003). At the time of writing, this genus included 20 species with validly published names (LPSN www.bacterio.net). As far as milk spoilage is concerned, a few species of *Brevibacillus* have been encountered in ‘commercially sterilized’ milk, raw milk, silage and milking equipment (Logan et al., 2002; Scheldeman et al., 2005). A polyphasic taxonomic study of aerobic endospore-forming bacteria by Logan et al. (2002) isolated two strains of *Brevibacilllus agri* (LMG 19651, LMG 19652) from samples of ‘commercially sterilized’ milk. Meanwhile, a molecular-based investigation (amplified ribosomal DNA restriction analysis, 16S rRNA gene sequencing, percent G+C content and DNA-DNA reassociations) into 17 dairy farms in diverse locations across Belgium indicated the presence of *B. agri* (4.8% of the total samples under investigation), *B. borstelensis* (7.2%) and *Brevibacillus* sp. (4.8%) in raw milk collected over a 5-months period in the winter (Scheldeman et al., 2005). Low levels of *B. borstelensis* (17 isolates from four samples) were also detected in whole milk and skim milk powders in a Chinese study looking at whole, skim and infant milk powders (Yuan et al., 2012). Hence, *Brevibacillus* is often encountered in milk and milk products globally, albeit at low levels.

The *Sporosarcina* genus, established by Kluyver and van Niel (1936), forms part of the family of the Bacillaceae. At the time of writing, the genus comprised 12 species with *Sporosarcina ureae* being the type species (Yoon et al., 2001; Reddy et al., 2003; An et al., 2007; Kwon et al., 2007; Yu et al., 2008; Tominaga et al., 2009; Kämpfer et al., 2010; Wolfgang et al., 2012). *Sporosarcina* originate from and are able to thrive in a variety of environments, e.g., soil, seawater, pond water in the Antarctic, human blood, raw bovine milk or factory equipment used in the manufacture of soy sauce (Yoon et al., 2001; Kwon et al., 2007; Yu et al., 2008; Tominaga et al., 2009; Wolfgang et al., 2012). Meanwhile, the *Paenisporosarcina* genus was established by Krishnamurthi et al. (2009). At the time of writing, this genus comprised of four species. An investigation into environmental samples collected from a New York State dairy farm, using a developed culture-dependent selection strategy and an *rpoB* sequence-based subtyping method, yielded only 0.2% of *Sporosarcina* sp. (Huck et al., 2008). Therefore, based on this study, the prevalence of *Sporosarcina* and *Paenisporosarcina* strains in foodstuffs and, more relevantly, milk, is not high when compared to other aerobic spore-formers.

## Spoilage of Milk and Dairy Products by *Bacillus* and Related Genera

Aerobic spore-forming bacteria are a major concern to the dairy industry, less for their pathogenicity but more for their spoilage-causing capabilities. Food safety and product quality are known to be affected by these microoorganisms by three different mechanisms – (i) production of toxins, (ii) production of spoilage enzymes, and (iii) affecting the production of secondary dairy products such as cheese, yogurt, and milk powders (De Jonghe et al., 2010). *B. cereus* is an example of a spore-former, commonly encountered in the dairy industry that is capable of causing food poisoning *via* the production of toxins as demonstrated in Section “*Bacillus cereus*.”

### Production of Spoilage Enzymes

Enzymes, indigenous or microbial, can either be beneficial or detrimental to the dairy industry. Raw milk originating from healthy udder cells already contains an array of indigenous enzymes (Andrews, 1991; Deeth and Fitz-Gerald, 1994). However, most of these enzymes do not affect food safety and product quality, with only proteases and lipases that extensively influence the value and quality of milk and milk products by impacting on sensory qualities such as texture, taste, aroma, and nutritional value (Teh et al., 2014).

On the positive side, indigenous milk proteases such as plasmin are essential for the manufacture of many cheeses while esterases or lipases impact on cheese flavor development by hydrolysing milk fat to free fatty acids (Somers and Kelly, 2002; Holland et al., 2005). Though enzymes produced by bacteria are mostly regarded as unfavorable, enzymes deriving from lactic acid bacteria (LAB) are of critical importance (Mensah et al., 1991) and influence sensory properties of products, especially flavor and texture in cheese and yogurt (Giraffa, 2014).

In contrast, spoilage enzymes encountered in the dairy industry originate from a range of bacteria, namely, *Pseudomonas* (Adams et al., 1975), *Clostridium, Arthrobacter, Microbacterium, Streptococcus, Corynebacterium* (Chen et al., 2003), *Flavobacterium, Acinetobacter* (Craven and Macauley, 1992) and most importantly, the order Bacillales which include the *Bacillus, Paenibacillus*, and *Viridibacillus* genera (Collins, 1981; Phillips et al., 1990; Moreno Switt et al., 2014). These microorganisms produce a variety of proteases, lipases, and phospholipases that impact on the texture of dairy products and cause typical off-flavors (Lücking et al., 2013). For example, *B. cereus* is known to cause off-flavors such as the ‘’bitty cream” and ‘’sweet curdling” as a result of its enzymatic activities. The ‘’sweet curdling” defect, i.e., the curdling of milk without the addition of any acidifying agent is caused by the action of proteolytic enzymes while the ‘’bitty cream” defect, which can be described as the floating of clumps of fat, is caused by lecithinase activity (Coorevits et al., 2010). Other off-flavors such as the bitter, fruity, or rancid defects as well as flat-sour spoilage are caused by the presence of an array of other microorganisms in pasteurized and intermittently, UHT milk. Consequently, bacterial enzymes are a major contributor to the spoilage of dairy products and hence, causing considerable economic losses.

### Production of Secondary Dairy Products

Milk products have been manufactured for millennia to maintain a longer shelf-life. However, the quality and safety of dairy products are heavily dependent on the initial quality of raw milk as the prevalence of any defect or pathogen will most likely be increased during processing. With the global increase in consumption and demand of dairy products, it is essential for dairy manufacturers to strictly adhere to rules and regulations to meet quality control standards and customer requirements. Therefore, the presence of spore-forming bacteria in raw milk poses a major problem with respect to the production of secondary dairy products as they can give rise to several structural, chemical, and organoleptic defects.

#### Yogurt

Yogurt is one of the most highly consumed dairy products with, for example, an average production of 1938 million kilograms and consumption of 6.2 kilograms per capita in the US in 2011 (Clark et al., 2014). Although the main contaminants of yogurts are yeasts and molds, bacterial contamination by spore-forming bacteria resistant to industrial heat treatments, can gradually lead to deterioration of quality (MacBean, 2009). Common defects encountered in yogurts include high acid levels that produce a high acetaldehyde flavor (Vedamuthu, 1992), defects related to appearance and texture and, most relevant to this review, the incorporation of bitter flavors in the end product caused by proteinase activities of spore-forming bacteria such as *B. cereus* or *B. subtilis* (Mistry, 2001).

#### Dairy Powders

In contrast, microbial growth in powdered dairy ingredients is minimal because of their low water content (Chen et al., 2003). They can, nevertheless, become contaminated with thermoduric or thermophilic spore-forming bacteria during the manufacturing process. The vegetative cells and spores of these bacteria are able to survive pasteurization and may go on to form biofilms on the stainless steel surfaces of heat exchangers and evaporators (Flint, 1998). Contamination of the powdered ingredients occur sporadically, when fragments of the cells or biofilms are sloughed off and enter the processing stream affecting both product quality and limiting the run length of the processing plant (Flint et al., 1997b; Hinton et al., 2002). Even though most thermophilic bacteria do not go on to cause diseases (Scott et al., 2007), given most suitable conditions the dormant spores can germinate, producing acids, and enzymes that impact on the composition and organoleptic properties of the powders (Chen et al., 2004; Flint et al., 2007). In extreme cases of spore contamination, concentrations of up to 10^5^/g of milk have been detected, which far exceed typical specifications resulting in lower-value out-of-specification products (Seale et al., 2008) that fail to meet customer requirements.

#### Infant Milk Formula

Infant milk formula is produced in such a way to mimic the composition of human milk in terms of nutrients and its production is regulated by the European Commission (1991) as well as the Codex Alimentarius Commission (2007). Powdered IMF is known to influence the development of the gut flora in newborn infants with sterile gastrointestinal tracts. Therefore, for the safety of infants, pathogens such as *S. aureus, B. cereus, C. perfringens*, Enterobacteriaceae (coliforms), and *Salmonella* are screened for in all IMF production batches (Mackie et al., 1999; Forsythe, 2005).

To date, several bacterial genera such as *Bacteroides, Bifidobacterium, Clostridia, Lactobacilli, Streptococci, Salmonella, Klebsiella*, and *Cronobacter* have been reported in IMF (Stark and Lee, 1982; Benno et al., 1984; Harmsen et al., 2000; Forsythe, 2005). However, the literature focuses mainly on *Cronobacter sakazakii*, an emergent pathogenic contaminant of IMF that causes a life-threatening form of neonatal meningitis, bacteraemia, necrotising enterocolitis, and necrotising meningoencephalitis in healthy as well as premature and immuno-compromised infants (Muytjens et al., 1983; Iversen and Forsythe, 2003; Walsh et al., 2011). As far as spore-forming bacteria are concerned, the incidence of *Bacillus* sp., albeit at low levels, has been reported in IMF. An Italian study by Di Pinto et al. (2013), identified 11 (out of 60) positive IMF samples for *Bacillus* sp. with five characterized as *B. cereus* and the remaining being *B. licheniformis, B. subtilis*, and *B. mycoides*. Meanwhile, Lequin et al. (2005) studied three pre-term infants who suffered fatal hemorrhagic meningoencephalitis due to *B. cereus* infections, acquired from unknown sources. Therefore, taking into consideration that IMF can effectively harbor pathogenic microorganisms, it is essential to detect and control these bacteria for the safety of infants and children.

#### Cheese

Cheese is one of the most traded dairy products globally, with more than 8.4 million tons produced in the EU in 2011 (O’Sullivan et al., 2013). Milk, rennet and salt are the only staple ingredients in cheese production, but a variety of microbial interactions with these ingredients allow for the production of approximately a thousand cheese varieties (Fox and McSweeney, 2004; Fox et al., 2004; O’Sullivan et al., 2013). As a result of the intricate interactions between these microorganisms, cheese is probably one of the secondary products of milk that is mostly affected by spoilage bacteria with a range of factors involved in determining the spoilage. Depending on the spoilage microorganisms present, common cheese defects include taste and odor defects, biogenic amine (BA) formation, gas formation, secondary fermentations, mineral deposition, and cheese pinking (O’Sullivan et al., 2013).

The main defect in cheese involving spore-forming bacteria is late gas blowing. The latter is usually seen in Dutch or Swiss-type cheeses when spores of butyric acid bacteria germinate during cheese ripening and anaerobically ferment lactate to acetate, butyrate, carbon dioxide, and hydrogen (Sheehan, 2011, 2013). The production of BAs is another defect encountered in cheese. High concentrations of BAs have been detected in fermented and non-fermented foods products since an array of species from different genera can decarboxylate one or more amino acids (Maijala et al., 1995; Marino et al., 2000). Nonetheless, more relevant to this review is the fact that a few strains of *Bacillus* (*B. subtilis, B. megaterium, B. licheniformis, B. amyloliquefaciens*) have amine-producing capabilities (Zaman et al., 2010). These BAs can cause toxic reactions that are detrimental to health. Amine production cheese is prevalent due the availability of free amino acids after proteolysis, the presence of decarboxylase-positive microorganisms and suitable environmental factors (Burgess et al., 2010).

In summary, milk is a highly nutritious but perishable food product that can easily harbor a range of spoilage organisms. While contamination with non-spore-forming bacteria post-pasteurization or due to inadequate heat treatments can easily be rectified, contamination with Gram positive thermophilic or psychrotolerant spore-forming bacteria cannot be eradicated due to their ability to survive industrial pasteurization regimens (Ivy et al., 2012). These spore-forming bacteria are abundant in dust, dairy-feed concentrates, silage and forages, therefore, easily colonize skin and hair of cattle and contaminate milk (Ledenbach and Marshall, 2010). Colonization of dairy manufacturing plants by *Bacillus* sp. and relatives give rise to a reservoir of contamination, in the form of biofilms, which result in spoilage and low quality products.

## Biofilms

The complex process of biofilm formation and dissemination is responsible for the transmission of spoilage as well as pathogenic bacteria in the form of detached clumps and clusters (Hall-Stoodley and Stoodley, 2005). Bacteria tend to form biofilms to allow survival in hostile environments. These microorganisms can attach to a wide range of surfaces as long as nutrients are available. Surfaces can include living tissues, in-dwelling medical devices, industrial or potable water system piping, stainless steel food processing equipment or natural aquatic systems (Donlan, 2002). Research has shown that biofilm formation is widespread in food and beverage industries due to the high availability of nutrients and organic components. Once these biofilms are established, they go on to cause substantial losses to industries in a variety of ways. Significantly, a number of food pathogens, including *Listeria monocytogenes, B.cereus* and *Salmonella* sp. are among those capable of forming biofilms (Chmielewski and Frank, 2003).

### The Effects of Biofilms in Dairy Processing Plants

Milk is an ideal culture medium for both spoilage and pathogenic microorganisms, which go on to contaminate milk processing lines and dairy products. These microorganisms can contaminate either in the form of vegetative cells, spores, or detached biofilm clumps that adhere to the stainless steel components. Processing lines in dairy manufacturing plant are generally made of stainless steel as it matches the requirements of materials that come in contact with food. Even though stainless steel is the ideal material to use for equipment in dairy manufacturing plants, the aggregation, adherence, and contamination by biofilms cannot be prevented. Studies have established that dead ends, corners, cracks, crevices, gaskets, valves, and joint are all suitable areas for biofilm formation and, therefore, it is essential to use surfaces with minimal cracks and crevices to reduce bacterial adherence and biofilm formation (Storgards et al., 1999a,b; Bremer et al., 2009).

The most common aerobic thermophilic/thermoduric spore-formers identified across dairy manufacturing plants, and dairy products, belong to the *Bacillus* and *Geobacillus* genera, with *B. licheniformis, B. coagulans, B. cereus, B. pumilus*, and *Geobacillus* sp. (mainly *G. stearothermophilus)* being frequently identified in dairy biofilms (Flint et al., 1997a; Murphy et al., 1999; Parkar et al., 2001; Scott et al., 2007; Burgess et al., 2010; Shaheen et al., 2010; Yuan et al., 2012). As noted above, *G. stearothermophilus* is a non-pathogenic, aerobic spore-forming bacterium that is commonly isolated from contaminated milk products, especially milk powders but is only present at low levels in raw milk (Murphy et al., 1999). Several studies have demonstrated that the bacterial adhesion process can also be influenced by additional secondary factors. For example, bacteria such as *B. cereus* require additional milk components such as natural surfactants and phospholipids to colonize stainless steel equipment as it has been shown that certain strains of *B. cereus* can form biofilms in whole milk but not in water-diluted milk, probably due to the presence of surface-active compounds found in whole milk that act as a surfactant in biofilm formation (Shaheen et al., 2010). However, with respect to protein fouling, milk proteins, and milk aggregates can contribute to the growth of adherent bacteria as a consequence of being ideal substrates for bacterial growth, giving high specific growth rates and having the ability to sustain bacterial populations for a long period of time (Yoo et al., 2006). Investigations involving several species of *Bacillus* have established that the spore form of the bacterium attaches more readily than vegetative cells to stainless steel components as they have a relatively high hydrophobicity, are able to resist heat and chemicals and have greater solid surfaces-adhering capabiities (Roberts and Hitchins, 1969; Rönner et al., 1990; Hüsmark and Rönner, 1992).

Once these biofilms have colonized the processing environments, it does not take long before they start to detach and disperse, leading to contamination of products. Biofilm detachment can occur by three different processes – (i) erosion that occurs due to fluid shear forces; (ii) abrasion, which is the result of collision of particles and (iii) sloughing, which can be defined as the instant loss of large parts or the complete biofilm (Morgenroth, 2003; Garny et al., 2008). Given that biofilms can cause such a variety of potential hazards for the dairy industry, it is essential that companies maintain good hygienic practices and comply with high customer specifications, stringent biofilm monitoring and control strategies (McGuire and Kirtley, 1989; Flint et al., 1997a; Shaheen et al., 2010).

### Monitoring of Biofilms

Detection, monitoring and control of biofilms in the dairy industry are vital to controlling spoilage of heat-treated products and, in the context of pathogenic microbes, ensuring consumer safety. To be able to minimize contamination and build-up of biofilms within equipment, adequate, and timely monitoring is of the essence. Biofilm detection and monitoring have, over the years, been performed using culture-dependent as well as culture-independent online methods. While a myriad of approaches are available, it has been highlighted that some of the methods developed in the laboratory to detect and monitor biofilms cannot directly be transferred to industrial settings due to their delicate nature or limited practicability (Janknecht and Melo, 2003).

The most common of the biofouling monitoring methods that are used by industry involve indirect testing through assessing process performance, product quality or off-line chemical, physical, and biological studies on samples collected from the manufacturing process (Flemming, 2003). However, other approaches exist which rely on qualitative visual inspection of equipment, direct measurement of microbiological characteristics of biofilms, heat transfer and pressure drop measurements across the manufacturing line, measurement of optical signals, measurement of electric signals, or measurement of vibration signals (Pereira et al., 2009). Biofilm detection has also been attempted by researchers and clinicians using a variety of molecular methods. These, and other, techniques are not discussed in detail in this review and readers are referred to several other reviews that specifically address the topic of biofouling monitoring (Flemming, 2003; Janknecht and Melo, 2003; Hasting, 2005; Pereira et al., 2009; Tan et al., 2014). Once accurate detection and monitoring approaches have been established, the next step is then to devise and apply reliable control measures.

### Control of Biofilms

As is apparent from the information provided above, the control and removal of biofilms is essential with respect to preventing the contamination of dairy products. It is also critical with respect to preventing mechanical blockages and failures in equipment. Several approaches can be used to control biofilms in the dairy industry and the most common ones are as follows: alteration of the surface chemistry to prevent cell attachment, treatment of surfaces with antimicrobial agents, optimization of the process and equipment design or the use of intensive cleaning regimens (Bower et al., 1996). In the latter case, cleaning-in-place (CIP) processes are particularly important. These involve the washing out of milk processing lines with cleaning and sanitizing chemicals, with additional antimicrobial substances for an enhanced performance. Although CIP has proven to be a valuable strategy since its introduction in the 1950s (Bremer et al., 2009), there have been efforts to develop new strategies based on increased understanding of bacterial genetics, systems biology, materials and mechanic engineering and chemical biology (Tan et al., 2014). Other strategies to control biofilms in the dairy industry have also been proposed. These include the so-called green strategy for biofilm control, namely enzyme-based detergents, control using bacteriophages and control through microbial interactions or metabolite molecules (Simões et al., 2010); disinfection and removal of biofilm layers using ozone (O_3_) water and hydrogen peroxide solution (Tachikawa and Yamanaka, 2014); ultrasound treatments coupled with heat and/or pressure (Piyasena et al., 2003); high-intensity focused ultrasound (Xu et al., 2012); *via* the usage of biotherapeutic agents such as lactoferrin (Ammons and Copié, 2014) and *via* the usage of a variety of antimicrobials such as nisin, lauricidin or reuterin (Dufour et al., 2004; El-Ziney and Jakobsen, 2009). Some of these approaches are described in greater depth below, and a summary of the benefits and limitations of the various approaches can also be found in **Table 1**.

**Table 1:** Benefits and limitations of approaches used to control and remove biofilms prevalent in the dairy industry.

Control strategies	Examples of approach	Pros	Cons	Reference
**Physical approach**				
(1) Cleaning-in-place processes (CIP)	*Via* a combination of acidic (e.g., nitric acid) and alkaline (e.g., sodium hydroxide) detergents	• Highly efficient if specifically optimized as per the fouled deposits being treated • Multi-functional systems are efficient and economic • Efficiency of regimes can be increased by altering conditions such as temperature and concentration of detergents	• High water consumption • High energy usage to operate equipment • Single-use CIP units are expensive to operate • Process is time-consuming and any cleaning time is classified as downtime	Palabiyik et al., 2015 Tamime, 2008
(1a) Chemically enhanced CIP	Specific CIP with the addition of surfactants, chelating compounds, emulsifying agents, or complexing agents	• Chemicals increase effectiveness of cleaning process • Provide bacteriostatic conditions to delay further growth of microorganisms	• Chemicals are non-biodegradable and not environmentally friendly • Increased costs • Requires post-cleaning neutralization	White and Rabe, 1970
(1b) Biologically enhanced CIP	Specific CIP with the addition of enzymes	• Reduced usage of potentially hazardous chemicals • More environmentally friendly than using chemicals • Reduced energy consumption as enzymes can operate at low temperatures • Enzymatic cleaning reduces chemical and thermal stress on cleaning equipment	• Commercial enzymatic preparations are expensive • Enzymatic activity can be reduced by the presence of *in situ* inhibitors	Paul et al., 2014
(2) Ultrasonication	Low frequency/high intensity ultrasound (≥20 kHz)	• Easily de-agglomerates bacterial clusters by acoustic cavitation • No loss of nutrients and flavors as ultrasonication is non-thermal	• Technique is expensive due to high intensities required • Not efficient enough on its own and requires combining with other treatment techniques such as enzymes or biocides	Joyce et al., 2003 Cameron et al., 2008 Srey et al., 2013
**Biological approach**				
(1) Biological biocides	Enzymes (e.g., proteases and polysaccharide-hydrolysing enzymes) and bacteriocins (e.g., nisin, lacticin 3147, or pediocin PA1)	• Environmentally friendly • Bacteriocins deriving from lactic acid bacteria have GRAS status (generally recognized as safe) • Wide antibacterial spectrum • Bacteriocins are not active and non-toxic to eukaryotic cells • Bacteriocins do not affect the gut microbiota	• Enzymes are highly specific and setting up a cocktail of enzyme against biofilms is time-consuming and expensive • Bacteria in biofilms have developed resistance to biocides • Bacteriocins do not always target the desired bacterial group • A complex food microbiota can reduce the efficacy of bacteriocins	Cleveland et al., 2001; Sobrino-López and Martín-Belloso, 2008 Gálvez et al., 2007
(2) Bacteriophages	Act *via* a lytic or lysogenic pathway to infect bacteria	• Highly specific to target pathogenic bacteria • Self-limiting • Self-dosing • Rapid isolation • Less costly than antibiotics	• Phage-resistance mechanisms are evolving • Some phages carry antibiotic-resistance genes and other virulence factors • Phages are perceived as intruders by the immune system (immunogenicity), which affect *in vivo* efficacy • Ethical and social concerns towards genetic manipulation	Kutter et al., 2010; Nobrega et al., 2015
**Chemical approach**				
(1) Chemical biocides	Oxidizing agents (e.g., hydrogen peroxide), chlorine-based detergents (e.g., sodium hypochlorite), surface active compounds (e.g., quaternary ammonium compounds), iodophores	• Chemical biocides work at a range of pH, temperature, and concentrations • More cost-efficient as compared to biological biocides • Chemical mixes are easily prepared and applied	• Not environmentally friendly • Chlorine-based detergents are instable and toxic • Some chemicals have corrosive effects on surfaces • Hydrogen peroxide is effective only at high concentrations	Van Houdt and Michiels, 2010 Vlková et al., 2008
(2) Ozone	*Via* either molecular ozone or its decomposition products (e.g., hydroxyl radicals)	• Environmentally friendly • Has GRAS status • Rapid inactivation and leaves no residual components • Low overall energy consumption	• Efficacy is highly dependent on temperature and pH as well as target microorganism • Can cause irritation and other symptoms even at low concentrations	Graham, 1997; Khadre et al., 2001; Cullen and Norton, 2012

#### Cleaning-in-Place Processes

Cleaning-in-place protocols vary according to industries and the residues that need to be cleaned. Within dairy manufacturing plants, deposits can either be organic such as fats, un-denatured and denatured proteins, sugar, minerals, fruit cells and secondary manufacturing products such as gums, starches, emulsifiers and stabilizers, or inorganic such as calcium phosphate (Walton, 2008). As some processed dairy products have lower nutrient concentrations and water activity as compared to raw milk, spore-formation is favored over growth of vegetative cells, enhancing the need for different cleaning regimes in processing plants. While the cleaning process involves a combination of aqueous, foam or gel acidic and alkaline detergents, disinfection is carried out *via* a range of biocides that are able to reduce live bacterial cells and their subsequent outgrowth on surfaces such that they do not affect the specification of products. In the dairy industry, alkaline/caustic detergents (e.g., sodium hydroxide) are effective against organic deposits such as milk proteins and fat and work best at a temperature of 60–73°C whilst acidic solutions (nitric acid) removes mineral deposits (e.g., calcium phosphate) from milk and water (Chisti, 1999; Keown and Kononoff, 2006; Bava et al., 2011). Nowadays, caustic blends containing additives, surfactants, emulsifying agents, chelating compounds and complexing agents and acid blends containing other acids and surfactants are being used for enhanced cleaning regimens and safer end products (Bremer et al., 2006).

#### Biocides

Even though optimized CIP processes are the standard cleaning procedures globally across dairy industries, the process mainly focuses on cleaning residues from equipment surfaces and reducing viable bacterial counts rather than removing established biofilms and reducing biofilm development (Parkar et al., 2003). As none of the current control strategies are able to remove biofilms completely, better and more effective biocides that are in line with the EC directive regarding biocidal products [Regulation (EU) No 528/2012], combined control processes and alternative control methods need to be implemented, while at the same time keeping in mind costs, labor, energy usage, and product safety (Simões et al., 2010).

Across the dairy industry, the use of chemical biocides is routine with the choice of biocide reflecting some important considerations, including the need to be environmentally friendly, economical, non-toxic, non-allergenic, should not react with the surface material (corrosion or staining), is stable over ranges of pH and temperatures and should have a broad activity spectrum (Møretrø et al., 2012). The most common chemical disinfectants include oxidizing agents and chlorine-based detergents (sodium hypochlorite), hydrogen peroxide, ozone and peracetic acid, surface active compounds including quaternary ammonium compounds (QACs) and acid anionic compounds and iodophores (Van Houdt and Michiels, 2010). Disinfection alone is not enough to eliminate biofilms as they leave the EPS matrix intact (Pontefract, 1991). Therefore, it has been suggested that chemical control of biofilms should be coupled with mechanical methods to disrupt and break down the matrix, hereby reducing bacterial adherence to surfaces and increasing the effects of chemical biocides (Kumar and Anand, 1998).

More recently, biological biocides such as enzymes and bacteriocins have gradually been introduced in an attempt to change the physico-chemical properties of adhesion surfaces as well as to provide a ‘greener’ and more environmentally friendly control method. Enzymes, which are non-toxic and highly specific, have been used by several research groups as an alternative method of control of biofilms. The efficiency of these enzymes is dependent on the species of bacteria involved and, as the EPS matrix is heterogeneous in nature, a combination of enzymes is usually required (Simões et al., 2010). When combined with surfactants to improve cleaning efficacy, proteases (e.g., savinase, everlase, polarzyme) and polysaccharide-hydrolysing enzymes (e.g., amylase, cellulase, pectinesterase, pectin lyase) have proven effective in the degradation of the EPS matrix and removal of bacterial biofilms (Parkar et al., 2004; Molobela et al., 2010; Srey et al., 2013). Meanwhile, bacteriocins, which are “ribosomally synthesized antimicrobial peptides produced by one bacterium that are active against bacteria, either in the same species (narrow spectrum) or across genera (broad spectrum)” (Cotter et al., 2005) have also been trialed in the control of biofilms (Bowdish et al., 2005). The LAB-derived bacteriocins that could potentially be employed by the dairy industry for this purpose include nisin, lacticin 3147 or 481, and pediocin PA-1 (Deegan et al., 2006). Notably, nisin is already widely used across the food industry and is categorized as a generally recognized as safe (GRAS) compound. These bio-active compounds can be adsorbed onto surfaces and are able to inhibit the adhesion of bacterial cells. There are thus a range of chemical and biological biocides which are relatively effective against biofilms. However, the development of resistance to some of the compounds already in use means that the more rapid commercialisation of solutions that to date have only been applied at a laboratory scale, is essential.

#### Other Methods

While CIP processes and biocides have been key components of conventional cleaning approaches in dairy manufacturing plants, the aforementioned acquisition of resistance by bacteria is a cause for concern. Therefore, novel and improved control approaches must be introduced to maintain the quality and safety of dairy products. A summary of a few emerging control strategies will be presented in this review and readers are directed to reviews dedicated to novel and emerging methods of biofilm control for a comprehensive insight into the subject (Simões et al., 2010; Teixeira and Rodrigues, 2014).

##### Ozone

Ozone, a highly oxidizing gas, is a potent antimicrobial used to combat bacteria, fungi, viruses, protozoa, and bacterial spores via the disruption of the cell envelope (Khadre et al., 2001; Srey et al., 2013). Khadre et al. (2001) have demonstrated that molecular ozone and its decomposition products, e.g., hydroxyl radicals, react with intracellular enzymes, nucleic materials and components of the cell envelope and spore coats to inactivate bacteria and, hence, disintegrate biofilms. However, despite having strong antimicrobial properties, ozone is unstable and quickly degenerates into its by-products, water and oxygen, before it can act on microorganisms (Teixeira and Rodrigues, 2014).

##### Ultrasonication

Ultrasonication is “a non-thermal process that uses sound waves with frequencies greater than the limit for human hearing, i.e., 20 kHz or higher” to inactivate pasteurization-resistant microorganisms (Khanal et al., 2014a). Inactivation of bacteria is done via low frequency ultrasound (also called high intensity or power ultrasound) applied in a liquid medium, whereby power levels of 10–1000 W/cm^2^ are generated resulting in cavitation (Raso et al., 1998; Villamiel et al., 2009). One of the latest studies on ultrasonication applied to thermoduric aerobic spore-formers has determined that high intensity power ultrasonication is able to inactivate microorganisms within 10 min and, when combined with pasteurization, all vegetative microbial cells are inactivated (Khanal et al., 2014b). The same research group has also been investigating the actions of ultrasonication on spores of *B. coagulans, B.licheniformis*, and *G. stearothermophilus*. Their studies determined that high intensity ultrasonication combined with pasteurization inactivated 65.74% of endospores present in non-fat milk while ultrasonication combined with a heat treatment at 80°C for 1 min inactivated 75.32% of endospores (Khanal et al., 2014b). Despite being a powerful tool as it can reach biofilms in cracks and crevices, ultrasonication alone cannot completely dislodge all biofilms. As noted above, perhaps through its use in combination with other antimicrobial agents (also including EDTA or ozonation), it can prove to be efficient (Baumann et al., 2009; Teixeira and Rodrigues, 2014).

##### Bacteriophage

Bacteriophage or ‘phage’ are ubiquitous “viruses that infect bacteria and are found in the same biosphere niches as their bacterial hosts” (Kutter and Sulakvelidze, 2005). Phage are able to control microbial biofilms in a natural, non-toxic and highly specific manner while being dependent on chemical composition and factors such as temperature, growth stage, media, and phage concentration (Sillankorva et al., 2004; Chaignon et al., 2007). Phage can be used against single-species but also against multi-species biofilms as they are able to target their specific host without binding to co-resident species (Sillankorva et al., 2010). It has been suggested that phage and bacteria can gradually come to co-exist in biofilms and, thus, combining phages with polysaccharide depolymerases or disinfectants may be vital for successful applications (Tait et al., 2002). It is important to note, however, that although phage therapy is considered to have considerable potential for such applications, several limitations still hinder its widespread usage as a reliable approach. These drawbacks include (i) the acquisition of resistance by mutated bacteria *via* mechanisms such as the blockage of phage adsorption, (ii) the prevalence of antibiotic-resistant genes or bacterial virulence factors in the phages that can be transduced to bacteria, (iii) questions regarding immunogenicity and effective *in vivo* usage of phages (iv) ethical and social concerns from the public in relation to genetic manipulation, and (v) appropriate classification and regulation of native phages due to their unconventionality (Gill and Hyman, 2010; Hyman and Abedon, 2010; Kutter et al., 2010; Drulis-Kawa et al., 2012; Vandamme and Miedzybrodzki, 2013; Nobrega et al., 2015).

## Conclusion

Profitability and reputation in the dairy industry are highly dependent on the quality and safety of the products generated. Pasteurization and refrigeration are the most important processes employed by industry to control pathogens and spoilage-causing microorganisms, but, their success can be restricted due to the existence of several bacterial species that are able to survive the industrial pasteurization process. Aerobic spore-forming bacteria, which form resistant biofilms, are able to survive these harsh conditions and result in dairy manufacturing plants suffering economic losses and shorter run times for equipment. Presenting a significant challenge for the dairy industry as well as the food/beverages industry as a whole, a greater understanding of the composition of biofilms and the factors that lead to their development is vital. Comprehensive research into the development of physical, chemical, and biological control strategies will eventually lead to new and innovative approaches to control biofilms. Meanwhile, whilst the novel approaches to controlling biofilms are still being evaluated, CIP approaches enhanced by chemical or biological biocides remain the most reliable cleaning strategies in the dairy industry.

## Author contributions

NG - MSc student. CH and PC - Supervisor and co-supervisor. PR, TB, and MF - Scientific contributors and involved in setting up grant and fundings.

## Conflict of Interest Statement

The authors declare that the research was conducted in the absence of any commercial or financial relationships that could be construed as a potential conflict of interest.

## References

[B1] Adams D. M. Barach J. T. Speck M. L. (1975). Heat resistant proteases produced in milk by psychrotrophic bacteria of dairy origin. *J. Dairy Sci.* 58 828–834. 10.3168/jds.S0022-0302(75)84645-5 237945

[B2] Agata N. Ohta M. Mori M. Isobe M. (1995). A novel dodecadepsipeptide, cereulide, is an emetic toxin of *Bacillus cereus*. *FEMS Microbiol. Lett.* 129 17–20. 10.1016/0378-1097(95)00119-p 7781985

[B3] Ahn J.-H. Kim B. Kim B.-Y. Kim S.-J. Song J. Kwon S.-W. (2014). *Paenibacillus cucumis* sp. nov. isolated from greenhouse soil. *J. Microbiol.* 52 460–464. 10.1007/s12275-014-4071-7 24871973

[B4] Ammons M. C. Copié V. (2014). Mini-review: Lactoferrin: a bioinspired, anti-biofilm therapeutic. *Biofouling* 29 443–455. 10.1080/08927014.2013.773317 23574002 PMC3648868

[B5] An S.-Y. Haga T. Kasai H. Goto K. Yokota A. (2007). *Sporosarcina saromensis* sp. nov., an aerobic endospore-forming bacterium. *Int. J. Syst. Evol. Microbiol.* 57 1868–1871. 10.1099/ijs.0.64962-0 17684272

[B6] Andersson A. Granum P. E. Rönner U. (1998). The adhesion of *Bacillus cereus* spores to epithelial cells might be an additional virulence mechanism. *Int. J. Food Microbiol.* 39 93–99. 10.1016/S0168-1605(97)00121-9 9562881

[B7] Andrews A. T. (1991). “Indigenous enzymes in milk,” in *Food Enzymology* ed. Fox P. F. (New York, NY: Elsevier Science Publishers) 54–61.

[B8] Anonymous (1997). *Bacillus cereus Determination in Foods. NKML Method No.* 67 4th Edn. Oslo: The Nordic Committee on Food Analysis.

[B9] Ash C. Farrow J. Wallbanks S. Collins M. (1991). Phylogenetic heterogeneity of the genus *Bacillus* revealed by comparative analysis of small-subunit-ribosomal RNA sequences. *Lett*. *Appl. Microbiol.* 13 202–206. 10.1111/j.1472-765X.1991.tb00608.x

[B10] Ash C. Priest F. G. Collins M. D. (1993). Molecular identification of rRNA group 3 bacilli (Ash, Farrow, Wallbanks and Collins) using a PCR probe test. Proposal for the creation of a new genus *Paenibacillus*. *Antonie Van Leeuwenhoek* 64 253–260. 10.1007/BF00873085 8085788

[B11] Banat I. M. Marchant R. Rahman T. J. (2004). *Geobacillus debilis* sp. nov., a novel obligately thermophilic bacterium isolated from a cool soil environment, and reassignment of *Bacillus pallidus* to *Geobacillus pallidus* comb. nov. *Int. J. Syst. Evol. Microbiol.* 54 2197–2201. 10.1099/ijs.0.63231-0 15545458

[B12] Baumann A. R. Martin S. E. Feng H. (2009). Removal of *Listeria monocytogenes* biofilms from stainless steel by use of ultrasound and ozone. *J. Food Protect.* 72 1306–1309. 10.4315/0362-028x-72.6.1306 19610346

[B13] Bava L. Zucali M. Sandrucci A. Brasca M. Vanoni L. Zanini L. (2011). Effect of cleaning procedure and hygienic condition of milking equipment on bacterial count of bulk tank milk. *J.* *Dairy Res.* 78 211–219. 10.1017/S002202991100001X 21371358

[B14] Beattie S. Williams A. (1999). Detection of toxigenic strains of *Bacillus cereus* and other *Bacillus* spp. with an improved cytotoxicity assay. *Lett. Appl. Microbiol.* 28 221–225. 10.1046/j.1365-2672.1999.00498.x 10196773

[B15] Beecher D. J. Schoeni J. L. Wong A. C. (1995). Enterotoxic activity of hemolysin BL from *Bacillus cereus*. *Infect*. *Immun.* 63 4423–4428. 10.1128/iai.63.11.4423-4428.1995 PMC173629 7591080

[B16] Benno Y. Sawada K. Mitsuoka T. (1984). The intestinal microflora of infants; composition of fecal flora in breast and bottle fed infants. *Pediatr*. *Infect. Dis. J.* 4:308. 10.1111/j.1348-0421.1984.tb00754.x 6513816

[B17] Blowey R. W. Edmondson P. (2010). “Bactoscan and total bacterial count chap. 10” in *Mastitis Control in Dairy Herds* ed. Hulbert S. 2nd Edn. Oxfordshire: CAB International.

[B18] Bowdish D. M. Davidson D. J. Hancock R. E. (2005). A re-evaluation of the role of host defence peptides in mammalian immunity. *Curr*. *Protein Pept. Sci.* 6 35–51. 10.2174/1389203053027494 15638767

[B19] Bower C. McGuire J. Daeschel M. (1996). The adhesion and detachment of bacteria and spores on food-contact surfaces. *Trends Food Sci. Technol.* 7 152–157. 10.1016/0924-2244(96)81255-6

[B20] Bremer P. J. Fillery S. McQuillan A. J. (2006). Laboratory scale Clean-In-Place (CIP) studies on the effectiveness of different caustic and acid wash steps on the removal of dairy biofilms. *Int. J. Food Microbiol.* 106 254–262. 10.1016/j.ijfoodmicro.2005.07.004 16216371

[B21] Bremer P. J. Seale B. Flint S. Palmer J. Fratamico P. Annous B. (2009). “Biofilms in dairy processing,” in *Biofilms in the Food and Beverage Industries* eds Fratamico P. M. Annous B. A. Guenther N. W. (Amsterdam: Elsevier) 96–431.

[B22] Buehner K. P. Anand S. Garcia A. (2014). Prevalence of thermoduric bacteria and spores on 10 Midwest dairy farms. *J. Dairy Sci.* 97 6777–6784. 10.3168/jds.2014-8342 25200773

[B23] Burgess S. A. Flint S. H. Lindsay D. (2013). Characterization of thermophilic bacilli from a milk powder processing plant. *J. Appl. Microbiol.* 116 350–359. 10.1111/jam.12366 24119100

[B24] Burgess S. A. Lindsay D. Flint S. H. (2010). *Thermophilic bacilli* and their importance in dairy processing. *Int*. *J. Food Microbiol.* 144 215–225. 10.1016/j.ijfoodmicro.2010.09.027 21047695

[B25] Caamaño-Antelo S. Fernández-No I. Böhme K. Ezzat-Alnakip M. Quintela-Baluja M. Barros-Velázquez J. (2015). Genetic discrimination of foodborne pathogenic and spoilage *Bacillus* spp. based on three housekeeping genes. *Food Microbiol.* 46 288–298. 10.1016/j.fm.2014.08.013 25475298

[B26] Cameron M. McMaster L. D. Britz T. J. (2008). Electron microscopic analysis of dairy microbes inactivate by ultrasound. *Ultrason Biochem.* 15 960–964. 10.1016/j.ultsonch.2008.02.012 18406653

[B27] Chaignon P. Sadovskaya I. Ragunah C. Ramasubbu N. Kaplan J. B. Jabbouri S. (2007). Susceptibility of staphylococcal biofilms to enzymatic treatments depends on their chemical composition. *Appl. Microbiol. Biotechnol.* 75 125–132. 10.1007/s00253-006-0790-y 17221196

[B28] Chapela M. Garrido-Maestu A. Cabado A. Yildiz F. (2015). Detection of foodborne pathogens by qPCR: a practical approach for food industry applications. *Cogent Food Agric.* 1 10.1080/23311932.2015.1013771

[B29] Chen L. Coolbear T. Daniel R. M. (2004). Characteristics of proteinases and lipases produced by seven *Bacillus* sp. isolated from milk powder production lines. *Int. Dairy J.* 14 495–504. 10.1016/j.idairyj.2003.10.006

[B30] Chen L. Daniel R. M. Coolbear T. (2003). Detection and impact of protease and lipase activities in milk and milk powders. *Int. Dairy J.* 13 255–275. 10.1016/S0958-6946(02)00171-1

[B31] Chisti Y. (1999). “Modern systems of plant cleaning,” in *Encyclopedia of Food Microbiol* eds Robinson R. Batt C. Patel P. (London: Academic press) 1806–1815. 10.1006/rwfm.1999.3020

[B32] Chmielewski R. Frank J. (2003). Biofilm formation and control in food processing facilities. *Comp. Rev. Food Sci. Food Soc.* 2 22–32. 10.1111/j.1541-4337.2003.tb00012.x 33451238

[B33] Christiansson A. Bertilsson J. Svensson B. (1999). *Bacillus cereus* spores in raw milk: factors affecting the contamination of milk during the grazing period. *J. Dairy Sci.* 82 305–314. 10.3168/jds.S0022-0302(99)75237-9 10068952

[B34] Clark S. Jung S. Lamsal B. (eds) (2014). *Food Processing: Principles and Applications* 2nd Edn. Hoboken, NJ: John Wiley & Sons.

[B35] Claus D. Berkeley R. C. W. (1986). “Genus Bacillus cohn 1872, 174 AL,” in *Bergey’s Manual of Systematic Bacteriology* Vol. 2 eds Boone G. G. David R. Castenholz R. W. (Baltimore: Williams & Wilkins) 1105–1139.

[B36] Clavel T. Carlin F. Lairon D. Schmitt P. (2004). Survival of *Bacillus cereus* spores and vegetative cells in acid media simulating human stomach. *J. Appl. Microbiol.* 97 214–219. 10.1111/j.1365-2672.2004.02292.x 15186458

[B37] Cleveland J. Montville T. J. Nes I. F. Chikindas M. L. (2001). Bacteriocins: safe natural antimicrobials for food preservation. *Int. J. Food Microbiol.* 71 1–20. 10.1016/S0168-1605(01)00560-8 11764886

[B38] Codex Alimentarius Commission (2007). Standard for infant formula and formulas for special medical purposes intended for infants. *CODEX STAN* 72.

[B39] Collins E. B. (1981). Heat resistant psychrotrophic microorganisms. *J. Dairy Sci.* 64 157–160. 10.3168/jds.S0022-0302(81)82543-X 6894936

[B40] Coorevits A. De Jonghe V. Vandroemme J. Van Landschoot A. Heyndrickx M. De Vos P. (2010). “How can the type of dairy farming influence the bacterial flora in milk?,” in *Organic Farming and Peanut Crops* eds Coorevits A. De Jonghe V. Vandroemme J. Van Landschoot A. Heyndrickx M. De Vos P. (Ghent: NOVA Science Publishers) 123–136.

[B41] Cotter P. D. Hill C. Ross R. P. (2005). Bacteriocins: developing innate immunity for food. *Nat*. *Rev. Microbiol.* 3 777–788. 10.1038/nrmicro1273 16205711

[B42] Craven H. Macauley B. (1992). Microorganisms in pasteurised milk after refrigerated storage. 1. Identification of types. *Aust. J. Dairy Technol.* 1 38–45.

[B43] Crielly E. Logan N. Anderton A. (1994). Studies on the *Bacillus flora* of milk and milk products. *J. Appl. Bacteriol.* 77 256–263. 10.1111/j.1365-2672.1994.tb03072.x 7989250

[B44] Cullen P. J. Norton T. (2012). “Ozone sanitisation in the food industry,” in *Ozone in Food Processing* 1st Edn eds O’Donnell C. Tiwari B. K. Cullen P. J. Rice G. (Hoboken, NJ: Blackwell Publishing).

[B45] Daane L. Harjono I. Barns S. Launen L. Palleron N. Häggblom M. (2002). PAH-degradation by *Paenibacillus* spp. and description of *Paenibacillus naphthalenovorans* sp. nov., a naphthalene-degrading bacterium from the rhizosphere of salt marsh plants. *Int. J. Syst. Evol. Microbiol.* 52 131–139. 10.1099/00207713-52-1-131 11837295

[B46] Damgaard P. Larsen H. Hansen B. Bresciani J. Jørgensen K. (1996). Enterotoxin-producing strains of *Bacillus thuringiensis* isolated from food. *Lett. Appl. Microbiol.* 23 146–150. 10.1111/j.1472-765X.1996.tb00051.x 8862018

[B47] Deegan L. H. Cotter P. D. Hill C. Ross P. (2006). Bacteriocins: biological tools for bio-preservation and shelf-life extension. *Int. Dairy J.* 16 1058–1071. 10.1016/j.idairyj.2005.10.026

[B48] Deeth H. C. Fitz-Gerald C. H. (1994). “Lipolytic enzymes and hydrolytic rancidity in milk and milk products,” in *Advanced Dairy Chemistry* 2nd Edn Vol. 2 eds McSweeney P. L. H. Fox P. F. (London: Chapman & Hall).

[B49] De Jonghe V. Coorevits A. De Block J. Van Coillie E. Grijspeerdt K. Herman L. (2010). Toxinogenic and spoilage potential of aerobic spore-formers isolated from raw milk. *Int. J. Food Microbiol.* 136 318–325. 10.1016/j.ijfoodmicro.2009.11.007 19944473

[B50] Dhakal R. Chauhan K. Seale R. B. Deeth H. C. Pillidge C. J. Powell I. B. (2013). Genotyping of dairy *Bacillus licheniformis* isolates by high resolution melt analysis of multiple variable number tandem repeat loci. *Food Microbiol.* 34 344–351. 10.1016/j.fm.2013.01.006 23541201

[B51] Dhakal R. Seale R. B. Deeth H. C. Craven H. Turner M. S. (2014). Draft genome comparison of representatives of the three dominant genotype groups of dairy *Bacillus licheniformis* strains. *Appl.* *Environ. Microbiol.* 80 3453–3462. 10.1128/AEM.00065-14 PMC4018868 24657871

[B52] Di Pinto A. Bonerba E. Bozzo G. Ceci E. Terio V. Tantillo G. (2013). Occurence of potentially enterotoxigenic *Bacillus cereus* in infant milk powder. *Eur. Food Res. Technol.* 237 275–279. 10.1007/s00217-013-1988-8

[B53] Donlan R. M. (2002). Biofilms: microbial life on surfaces. *Emerg.* *Infect. Dis.* 8 881–890. 10.3201/eid0809.020063 PMC2732559 12194761

[B54] Doyle C. J. Gleeson D. Jordan K. Beresford T. P. Ross R. P. Fitzgerald G. F. (2015). Anaerobic sporeformers and their significance with respect to milk and dairy products. *Int. J. Food Microbiol.* 197 77–87. 10.1016/j.ijfoodmicro.2014.12.022 25574847

[B55] Drulis-Kawa Z. Majkowska-Skrobek G. Maciejewska B. Delattre A.-S. Lavigne R. (2012). Learning from bacteriophages – advantages and limitations of phage and phage-encoded protein applications. *Curr. Protein Peptide Sci.* 13 699–722. 10.2174/138920312804871193 23305359 PMC3594737

[B56] Dufour M. Simmonds R. S. Bremer P. J. (2004). Development of a laboratory scale Clean-In-Place system to test the effectiveness of “natural” antimicrobials against dairy biofilms. *J. Food Protect.* 67 1438–1443. 10.4315/0362-028x-67.7.1438 15270498

[B57] Durak M. Z. Fromm H. I. Huck J. R. Zadoks R. N. Boor K. J. (2006). Development of molecular typing methods for *Bacillus* spp. and *Paenibacillus* spp. isolated from fluid milk products. *J. Food Sci.* 71 M50–M56. 10.1111/j.1365-2621.2006.tb08907.x

[B58] Ehling-Schulz M. Vukov N. Schulz A. Shaheen R. Andersson M. Martlbauer E. (2005). Identification and partial characterization of the nonribosomal peptide synthetase gene responsible for cereulide production in emetic *Bacillus cereus*. *Appl. Environ. Microbiol.* 71 105–113. 10.1128/AEM.71.1.105-113.2005 15640177 PMC544239

[B59] El-Ziney M. G. Jakobsen M. (2009). Effectiveness of reuterin alone and in combination with nisin or other food contact surfaces sanitizers and cleaners for disinfection of stainless steel surfaces contaminated with *Escherichia coli* and *Listeria innocua*. *J. Food Agric. Environ.* 7 145–149.

[B60] European Commission (1991). Directive of 14 May 1991 on infant formulae and follow-on formulae. *Food and Drug Administration, O.J. No. L* 75 35.

[B61] European Commission (2014). *Development of the Dairy Market Situation and the Operation of the ‘Milk Package’ Provisions.* Report from the Commission to the European Parliament and the Council, Brussels. Available at: http://ec.europa.eu/agriculture/milk/milk-package/index_en.htm

[B62] European Food Safety Authority (2005). Opinion of the scientific panel on biological hazards on *Bacillus cereus* and other *Bacillus* spp. *Foodstuffs* 175 1–48.

[B63] Flemming H. (2003). Role and levels of real-time monitoring for successful anti-fouling strategies - an overview. *Water Sci. Technol.* 47 1–8. 12701899

[B64] Flint S. (1998). *Formation and Control of Biofilms of Thermo-Resistant Streptococci on Stainless Steel* thesis Department of Food Technology, Massey University Palmerston North.

[B65] Flint S. Bremer P. Brooks J. (1997a). Biofilms in dairy manufacturing plant-description, current concerns and methods of control. *Biofouling* 11 81–97. 10.1080/08927019709378321

[B66] Flint S. Brooks J. Bremer P. (1997b). “The influence of cell surface properties of thermophilic streptococci on attachment to stainless steel. *J. Appl. Microbiol.* 83 508–517. 10.1046/j.1365-2672.1997.00264.x 9351231

[B67] Flint S. Walker K. Waters B. Crawford (2007). Description and validation of a rapid (1 h) flow cytometry test for enumerating thermophilic bacteria in milk powders. *J. Appl. Microbiol.* 102 909–915. 17381733 10.1111/j.1365-2672.2006.03167.x

[B68] Forsythe S. J. (2005). *Enterobacter* sakazakii and other bacteria in powdered infant milk formula. *Matern Child Nutr.* 1 44–50. 10.1111/j.1740-8709.2004.00008.x 16881878 PMC6874386

[B69] Fox P. F. McSweeney P. L. (2004). “Cheese: an overview,” in *Cheese: Chemistry, Physics and Microbiology, General Aspects* 3rd Edn Vol. 1 eds Fox P. F. Paul L. H. McSweeney T. M. C. Guinee T. P. (London: Elsevier Academic Press) 1–18.

[B70] Fox P. F McSweeney P. L. H. Cogan T. M. Guinee T. P. (eds) (2004). *Cheese: Chemistry, Physics and Microbiology: General Aspects* 3rd Edn Vol. 1 (London: Elsevier Academic Press).

[B71] From C. Hormazábal V. Granum P. E. (2007). Food poisoning associated with pumilacidin-producing *Bacillus pumilus* in rice. *Int*. *J. Food Microbiol.* 115 319–324. 10.1016/j.ijfoodmicro.2006.11.005 17275116

[B72] From C. Pukall R. Schumann P. Hormazábal V. Granum P. E. (2005). Toxin-producing ability among *Bacillus* spp. outside the *Bacillus cereus* group. *Appl. Environ. Microbiol.* 71 1178–1183. 10.1128/AEM.71.3.1178-1183.2005 15746316 PMC1065142

[B73] Fromm H. I. Boor K. (2004). Characterization of pasteurized fluid milk shelf-life attributes. *J. Food Sci.* 69 M207–M214. 10.1111/j.1365-2621.2004.tb09889.x

[B74] Galperin M. Y. (2013). Genome diversity of spore-forming Firmicutes. *Microbiol. Spectr.* 1:TBS-0015-2012 10.1128/microbiolspectrum.TBS-0015-2012 PMC4306282 26184964

[B75] Gálvez A. Abriouel H. López R. L. Omar N. B. (2007). Bacteriocin-based strategies for food preservation. *Int. J. Food Microbiol.* 120 51–70. 10.1016/j.ijfoodmicro.2007.06.001 17614151

[B76] Garny K. Horn H. Neu T. R. (2008). Interaction between biofilm development, structure and detachment in rotating annular reactors. *Bioproc. Biosyst. Eng.* 31 619–629. 10.1007/s00449-008-0212-x 18320233

[B77] Ghelardi E. Celandroni F. Salvetti S. Barsotti C. Baggiani A. Senesi S. (2002). Identification and characterization of toxigenic *Bacillus cereus* isolates responsible for two food-poisoning outbreaks. *FEMS Microbiol. Lett.* 208 129–134. 10.1111/j.1574-6968.2002.tb11072.x 11934506

[B78] Gilbert R. J. Kramer J. M. (1984). *Bacillus cereus* enterotoxins: present status. *Biochem. Soc.* 12 198–200. 10.1042/bst0120198 6427037

[B79] Gilbert R. J. Kramer J. M. (1986). “*Bacillus cereus* food poisoning,” in *Proceedings of Symposium Progress in Food Safety* eds Cliver D. O. Cochrane B. A. (Mysore: Food Research Institute) 85–93.

[B80] Gill J. Hyman P. (2010). Phage choice, isolation and preparation for phage therapy. *Curr. Pharm. Biotechnol.* 11 2–14. 10.2174/138920110790725311 20214604

[B81] Giraffa G. (2014). “Overview of the ecology and biodiversity of the LAB,” in *Lactic Acid Bacteria – Biodiversity and Taxonomy* 1st Edn eds Holzapfel W. H. Wood B. J. B. (Chichester: John Wiley & Sons, Ltd.).

[B82] Graham D. M. (1997). Use of ozone for food processing. *Food Technol. Chic.* 51 72–75.

[B83] Granum P. E. Brynestad S. O’sullivan K. Nissen H. (1993). Enterotoxin from *Bacillus cereus*: production and biochemical characterization. *Ned. Melk Zui.* 47 63–70.

[B84] Granum P. E. O’Sullivan K. Lund T. (1999). The sequence of the non-haemolytic enterotoxin operon from *Bacillus cereus*. *FEMS Microbiol. Lett.* 177 225–229. 10.1016/S0378-1097(99)00312-2 10474188

[B85] Griffiths M. W. (1992). *Bacillus cereus* in liquid milk and other milk products. *Bull. Int. Dairy Fed.* 36–39.

[B86] Hall-Stoodley L. Stoodley P. (2005). Biofilm formation and dispersal and the transmission of human pathogens. *Trends Microbiol.* 13 7–10. 10.1016/j.tim.2004.11.004 15639625

[B87] Hammer P. Lembke F. Suhren G. Heeschen W. (1995). Characterization of a heat resistant mesophilic *Bacillus* species affecting quality of UHT-milk: a preliminary report. *Kieler Milchwirtsch. Forschungsber.* 47 297–305.

[B88] Harmsen H. J. Wildeboer-Veloo A. C. Grijpstra J. Knol J. Degener J. E. Welling G. W. (2000). Development of 16S rRNA-based probes for the coriobacterium group and the atopobium cluster and their application for enumeration of coriobacteriaceae in human feces from volunteers of different age groups. *Appl.* *Environ. Microbiol.* 66 4523–4527. 10.1128/AEM.66.10.4523-4527.2000 PMC92335 11010909

[B89] Hasting A. P. M. (2005). “Improving the monitoring of fouling, cleaning and disinfection in closed process plant,” in *Handbook of Hygiene Control in the Food Industry* eds Lelieveld H. L. M. Mostert M. A. Holah J. (Cambridge: Woodhead Publishing) 572–587.

[B90] Heyndrickx M. Coorevits A. Scheldeman P. Lebbe L. Schumann P. Rodriguez-Diaz M. (2012). Emended descriptions of *Bacillus sporothermodurans* and *Bacillus oleronius* with the inclusion of dairy farm isolates of both species. *Int*. *J. Syst. Evol. Microbiol.* 62(Pt 2) 307–314. 10.1099/ijs.0.026740-0 21398506

[B91] Heyndrickx M. Scheldeman P. (2002). “Bacilli associated with spoilage in dairy products and other food,” in *Applications and Systematics of Bacillus and Relatives* (Hoboken, NJ: Wiley) 64–82. 10.1002/9780470696743.ch6

[B92] Hillerton J. E. Berry E. A. (2004). “Quality of the milk supply: european regulations versus practice,” in *Proceeding of the NMC Annual Meeting* Compton 207–214.

[B93] Hinton A. Trinh K. Brooks J. Manderson G. (2002). Thermophile survival in milk fouling and on stainless steel during cleaning. *Food Bioprod. Process.* 80 299–304. 10.1205/096030802321154817

[B94] Holland R. Liu S.-Q. Crow V. Delabre M.-L. Lubbers M. Bennett M. (2005). Esterases of lactic acid bacteria and cheese flavour: milk fat hydrolysis, alcoholysis and esterification. *Int. Dairy J.* 15 711–718. 10.1016/j.idairyj.2004.09.012

[B95] Hong H. A. To E. Fakhry S. Baccigalupi L. Ricca E. Cutting S. M. (2009). Defining the natural habitat of *Bacillus* spore-formers. *Res. Microbiol.* 160 375–379. 10.1016/j.resmic.2009.06.006 19589385

[B96] Hoton F. M. Fornelos N. N’guessan E. Hu X. Swiecicka I. Dierick K. (2009). Family portrait of *Bacillus cereus* and *Bacillus weihenstephanensis* cereulide-producing strains. *Environ. Microbiol.* 1 177–183. 10.1111/j.1758-2229.2009.00028.x 23765791

[B97] Huck J. R. Sonnen M. Boor K. J. (2008). Tracking heat-resistant, cold-thriving fluid milk spoilage bacteria from farm to packaged product. *J. Dairy Sci.* 91 1218–1228. 10.3168/jds.2007-0697 18292280

[B98] Huck J. R. Woodcock N. H. Ralyea R. D. Boor K. J. (2007). Molecular subtyping and characterization of psychrotolerant endospore-forming bacteria in two New York State fluid milk processing systems. *J. Food Protect.* 70 2354–2364. 10.4315/0362-028x-70.10.2354 17969618

[B99] Hüsmark U. Rönner U. (1992). The influence of hydrophobic, electrostatic and morphologic properties on the adhesion of *Bacillus spores*. *Biofouling* 5 335–344. 10.1080/08927019209378253

[B100] Hyman P. Abedon S. T. (2010). Bacteriophage host range and bacterial resistance. *Adv. Appl. Microbiol.* 70 217–248. 10.1016/S0065-2164(10)70007-1 20359459

[B101] International Dairy Federation [Idf] (2013). *The Economic Important of Dairying.* Brussels: IDF Factsheet Publisher.

[B102] Iversen C. Forsythe S. (2003). Risk profile of *Enterobacter sakazakii*, an emergent pathogen associated with infant milk formula. *Trends Food Sci. Technol.* 14 443–454. 10.1016/S0924-2244(03)00155-9

[B103] Ivy R. A. Ranieri M. L. Martin N. H. den Bakker H. C. Xavier B. M. Wiedmann M. (2012). Identification and characterization of psychrotolerant sporeformers associated with fluid milk production and processing. *Appl. Environ. Microbiol.* 78 1853–1864. 10.1128/AEM.06536-11 22247129 PMC3298126

[B104] Jackson S. Goodbrand R. Ahmed R. Kasatiya S. (1995). *Bacillus cereus* and *Bacillus thuringiensis* isolated in a gastroenteritis outbreak investigation. *Lett*. *Appl. Microbiol.* 21 103–105. 10.1111/j.1472-765X.1995.tb01017.x 7639990

[B105] Janknecht P. Melo L. F. (2003). Online biofilm monitoring. *Rev. Environ. Sci. Biotechnol.* 2 269–283. 10.1023/B:RESB.0000040461.69339.04

[B106] Joyce E. Phull S. S. Lorimer J. P. Mason T. J. (2003). The development and evaluation of ultrasound for the treatment of bacterial suspensions. A study of frequency, power and sonication time on cultured *Bacillus* species. *Ultrason Sonochem* 10 315–318. 10.1016/S1350-4177(03)00101-9 12927605

[B107] Kalogridou-Vassiliadou D. (1992). Biochemical activities of *Bacillus* species isolated from flat sour evaporated milk. *J*. *Dairy Sci.* 75 2681–2686. 10.3168/jds.S0022-0302(92)78030-8 1430475

[B108] Kämpfer P. Falsen E. Lodders N. Schumann P. (2010). *Sporosarcina contaminans* sp. nov. and *Sporosarcina thermotolerans* sp. nov., two endospore-forming species. *Int. J. Syst. Evol. Microbiol.* 60 1353–1357. 10.1099/ijs.0.014423-0 19667369

[B109] Keown J. F. Kononoff P. J. (2006). *Producing Milk with a Low Bacteria Count.* (NebGuide: Institute of Agricultural and Natural Resources G) 678.

[B110] Khadre M. A. Yousef A. E. Kim J. G. (2001). Microbiological aspects of ozone applications in food: a review. *J. Food Sci.* 66 1242–1252. 10.1016/j.jviromet.2008.06.004

[B111] Khanal S. N. Anand S. Muthukumarappan K. (2014a). Evaluation of high-intensity ultrasonication for the inactivation of endospores of 3 bacilli species in nonfat milk. *J*. *Dairy Sci.* 97 5952–5963. 10.3168/jds.2014-7950 25087024

[B112] Khanal S. N. Anand S. Muthukumarappan K. Huegli M. (2014b). Inactivation of thermoduric aerobic sporeformers in milk by ultrasonication. *Food control* 37 232–239. 10.1016/j.foodcont.2013.09.022

[B113] Kim B. Bang J. Kim H. Kim Y. Kim B.-S. Beuchat L. R. (2014). *Bacillus cereus* and *Bacillus thuringiensis* spores in Korean rice: prevalence and toxin production as affected by production area and degree of milling. *Food Microbiol.* 42 89–94. 10.1016/j.fm.2014.02.021 24929722

[B114] Klijn N. Herman L. Langeveld L. Vaerewijck M. Wagendorp A. A. Huemer I. (1997). Genotypical and phenotypical characterization of *Bacillus sporothermodurans* strains, surviving UHT sterilisation. *Int. Dairy J.* 7 421–428. 10.1016/S0958-6946(97)00029-0

[B115] Kluyver A. J. van Niel C. B. (1936). Prospects for a natural system of classification of bacteria. *Zentralbl. Bakteriol. Parasitenk. Infektionskr. Hyg. Abt.* 94 369–403.

[B116] Kramer J. M. Gilbert R. J. (1989). *Bacillus cereus* and other *Bacillus* species. *Foodborne Bact Pathog.* 19 21–70.

[B117] Krishnamurthi S. Bhattacharya A. Mayilraj S. Saha P. Schumann P. Chakrabarti T. (2009). Description of *Paenisporosarcina quisquiliarum* gen. nov., sp. nov., and reclassification of *Sporosarcina macmurdoensis* Reddy et al., 2003 as *Paenisporosarcina macmurdoensis* comb. nov. *Int. J. Syst. Evol. Microbiol.* 59(Pt 6) 1364–1370. 10.1099/ijs.0.65130-0 19502317

[B118] Kuisiene N. Raugalas J. Chitavichius D. (2004). *Geobacillus lituanicus* sp. nov. *Int. J. Syst. Evol. Microbiol.* 54 1991–1995. 10.1099/ijs.0.02976-0 15545423

[B119] Kumar C. G. Anand S. (1998). Significance of microbial biofilms in food industry: a review. *Int*. *J. Food Microbiol.* 42 9–27. 10.1016/S0168-1605(98)00060-9 9706794

[B120] Kutter E. De Vos D. Gvasalia G. Alavidze Z. Gogokhia L. Kuhl S. (2010). Phage therapy in clinical practice: treatment of human infections. *Curr. Pharm. Biotechnol.* 11 69–86. 10.2174/138920110790725401 20214609

[B121] Kutter E. Sulakvelidze A. (eds) (2005). *Introduction, Bacteriophages: Biology, Applications* (Boca Raton, FL: CRC Press).

[B122] Kwon S.-W. Kim B.-Y. Song J. Weon H.-Y. Schumann P. Tindall B. J. (2007). *Sporosarcina koreensis* sp. nov. and *Sporosarcina soli* sp. nov., isolated from soil in Korea. *Int. J. Syst. Evol. Microbiol.* 57 1694–1698. 10.1099/ijs.0.64352-0 17684239

[B123] Law J. W. F. Ab Mutalib N. S. Chan K. G. Lee L. H. (2015). Rapid methods for the detection of foodborne bacterial pathogens: principles, applications, advantages and limitations. *Front. Microbiol.* 5:770 10.3389/fmicb.2014.00770 PMC4290631 25628612

[B124] Ledenbach L. H. Marshall R. T. (2010). “Microbiological spoilage of dairy products,” in *Compendium of the Microbiological Spoilage of Foods and Beverages* eds Sperber W. H. Doyle M. P. (New York, NY: Springer) 41–67.

[B125] Lemes A. R. N. Marucci S. C. Costa J. R. Alves E. C. Fernandes O. A. Lemos M. V. F. (2015). Selection of *B. thuringiensis* strains containing genes effective in the control of *Spodoptera frugiperda*. *Bt Res.* 6 1–8.

[B126] Lequin M. H. Vermeulen J. R. van Elburg R. M. Barkhof F. Kornelisse R. F. Swarte R. (2005). *Bacillus cereus* meningoencephalitis in preterm infants: neuroimaging characteristics. *Am*. *J. Neuroradiol.* 26 2137–2143. PMC8148855 16155172

[B127] Lindsay D. Mosupye F. Brözel V. Von Holy A. (2000). Cytotoxicity of alkaline-tolerant dairy-associated *Bacillus* spp. *Lett*. *Appl. Microbiol.* 30 364–369. 10.1046/j.1472-765x.2000.00732.x 10792664

[B128] Logan N. A. (2012). *Bacillus* and relatives in foodborne illness. *J. Appl. Microbiol.* 112 417–429. 10.1111/j.1365-2672.2011.05204.x 22121830

[B129] Logan N. A. De Vos P. (2009). “Genus IV. Brevibacillus shida, takagi, kadowaki and komagata 1996a, 942VP,” in *Bergey’s Manual of Systematic Bacteriology* Vol. 6 eds Garrity G. Boone D. R. Castenholz R. W. (New York, NY: Springer-Verlag) 304–316.

[B130] Logan N. A. Forsyth G. Lebbe L. Goris J. Heyndrickx M. Balcaen A. (2002). Polyphasic identification of *Bacillus* and *Brevibacillus* strains from clinical, dairy and industrial specimens and proposal of *Brevibacillus invocatus* sp. nov. *Int. J. Syst. Evol. Microbiol.* 52(Pt 3) 953–966. 10.1099/00207713-52-3-953 12054263

[B131] Lücking G. Stoeckel M. Atamer Z. Hinrichs J. Ehling-Schulz M. (2013). Characterization of aerobic spore-forming bacteria associated with industrial dairy processing environments and product spoilage. *Int. J. Food Microbiol.* 166 270–279. 10.1016/j.ijfoodmicro.2013.07.004 23973839

[B132] Lukášová J. Vyhnálková J. Páčová Z. (2001). *Bacillus* species in raw milk and in the farm environment. *Milchwissenschaft* 56 609–611.

[B133] Lund T. De Buyser M. L. Granum P. E. (2000). A new cytotoxin from *Bacillus cereus* that may cause necrotic enteritis. *Mol. Microbiol.* 38 254–261. 10.1046/j.1365-2958.2000.02147.x 11069652

[B134] MacBean R. D. (2009). “Packaging and the shelf life of yogurt,” in *Food Packaging and Shelf Life: A Practical Guide* ed. Robertson G. L. (Boca Raton, FL: CRC Press) 143.

[B135] Mackie R. I. Sghir A. Gaskins H. R. (1999). Developmental microbial ecology of the neonatal gastrointestinal tract. *Am. J. Clin. Nutr.* 69 1035s–1045s. 10232646 10.1093/ajcn/69.5.1035s

[B136] Magnusson M. Christiansson A. Svensson B. (2007). *Bacillus cereus* spores during housing of dairy cows: factors affecting contamination of raw milk. *J. Dairy Sci.* 90 2745–2754. 10.3168/jds.2006-754 17517714

[B137] Maijala R. Nurmi E. Fischer A. (1995). Influence of processing temperature on the formation of biogenic amines in dry sausages. *Meat Sci.* 39 9–22. 10.1016/0309-1740(95)80003-4 22059759

[B138] Mandal P. K. Biswas A. K. Choi K. Pal U. K. (2011). Methods for rapid detection of foodborne pathogens: an overview. *Am. J. Food Technol.* 6 87–102. 10.3923/ajft.2011.87.102

[B139] Marchant R. Banat I. (2010). “The genus Geobacillus and hydrocarbon utilization,” in *Handbook of Hydrocarbon and Lipid Microbiology* eds Timmis K. N. McGenity T. J. van der Meer J. R. de Lorenzo V. (Berlin: Springer) 1887–1896.

[B140] Marino M. Maifreni M. Moret S. Rondinini G. (2000). The capacity of *Enterobacteriaceae* species to produce biogenic amines in cheese. *Lett. Appl. Microbiol.* 31 169–173. 10.1046/j.1365-2672.2000.00783.x 10972722

[B141] Marquer P. (2013). *Portrait of the EU Milk Production Sector.* Eurostat: Statistics in focus Available at: http://ec.europa.eu/eurostat/statistics-explained/index.php/Archive:Milk_and_dairy_production_statistics

[B142] McGuire J. Kirtley S. A. (1989). On surface characterization of materials targeted for food contact. *J. Food Sci.* 54 224–226. 10.1111/j.1365-2621.1989.tb08609.x

[B143] Mensah P. Tomkins A. M. Drasar B. S. Harrison T. J. (1991). Antimicrobial effect of fermented *Ghanaian maize* dough. *J. Appl. Bacteriol.* 70 203–210. 10.1111/j.1365-2672.1991.tb02925.x 2030095

[B144] Mikkola R. Andersson M. A. Grigoriev P. Teplova V. V. Saris N.-E. L. Rainey F. A. (2004). *Bacillus amyloliquefaciens* strains isolated from moisture-damaged buildings produced surfactin and a substance toxic to mammalian cells. *Arch*. *Microbiol.* 181 314–323. 10.1007/s00203-004-0660-x 15014930

[B145] Mikkola R. Andersson M. A. Teplova V. Grigoriev P. Kuehn T. Loss S. (2007). Amylosin from *Bacillus amyloliquefaciens*, a K^+^ and Na^+^ channel-forming toxic peptide containing a polyene structure. *Toxicon* 49 1158–1171. 10.1016/j.toxicon.2007.02.010 17391722

[B146] Mikkola R. Kolari M. Andersson M. A. Helin J. Salkinoja-Salonen M. S. (2000). Toxic lactonic lipopeptide from food poisoning isolates of *Bacillus licheniformis*. *Eur. J. Biochem.* 267 4068–4074. 10866808 10.1046/j.1432-1033.2000.01467.x

[B147] Mikkola R. Saris N. E. Grigoriev P. A. Andersson M. A. Salkinoja-Salonen M. S. (1999). Ionophoretic properties and mitochondrial effects of cereulide: the emetic toxin of *B. cereus*. *Eur. J. Biochem.* 263 112–117. 10.1046/j.1432-1327.1999.00476.x 10429194

[B148] Mistry V. V. (2001). “Fermented milks and cream,” in *Applied Dairy Microbiology* 2nd Edn eds Marth E. H. Steele J. L. (New York, NY: Marcel Dekker Inc.) 301–326.

[B149] Moeller R. Horneck G. Rabbow E. Reitz G. Meyer C. Hornemann U. (2008). Role of DNA protection and repair in resistance of *Bacillus* subtilis spores to ultrahigh shock pressures simulating hypervelocity impacts. *Appl. Environ. Microbiol.* 74 6682–6689. 10.1128/AEM.01091-08 18791028 PMC2576695

[B150] Molobela I. P. Cloete T. E. Beukes M. (2010). Protease and amylase enzymes for biofilm removal and degradation of extracellular polymeric substances (EPS) produced by *Pseudomonas fluorescens* bacteria. *Afr. J. Microbiol. Res.* 4 1515–1524.

[B151] Moreno Switt A. I. Andrus A. D. Ranieri M. L. Orsi R. H. Ivy R. den Bakker H. C. (2014). Genomic comparison of sporeforming bacilli isolated from milk. *BMC Genomics* 15:26 10.1186/1471-2164-15-26 PMC3902026 24422886

[B152] Møretrø T. Heir E. Nesse L. L. Vestby L. K. Langsrud S. (2012). Control of *Salmonella* in food related environments by chemical disinfection. *Food Res. Int.* 45 532–544. 10.1016/j.foodres.2011.02.002

[B153] Morgenroth E. (2003). “Detachment: an often-overlooked phenomenon in biofilm research and modeling,” in *Biofilms in Wastewater Treatment* eds Wuertz S. Wilderer P. A. Bishop P. L. (London: IWA Publishing) 246–290.

[B154] Murphy P. M. Lynch D. Kelly P. M. (1999). Growth of thermophilic spore forming bacilli in milk during the manufacture of low heat powders. *Int. J. Dairy Technol.* 52 45–50. 10.1111/j.1471-0307.1999.tb02069.x

[B155] Muytjens H. Zanen H. Sonderkamp H. Kollee L. Wachsmuth I. K. Farmer J. (1983). Analysis of eight cases of neonatal meningitis and sepsis due to *Enterobacter sakazakii*. *J. Clin. Microbiol.* 18 115–120. 6885983 10.1128/jcm.18.1.115-120.1983 PMC270753

[B156] Nazina T. Tourova T. Poltaraus A. Novikova E. Grigoryan A. Ivanova A. (2001). Taxonomic study of aerobic thermophilic bacilli: descriptions of *Geobacillus subterraneus* gen. nov., sp. nov. and *Geobacillus uzenensis* sp. nov. from petroleum reservoirs and transfer of *Bacillus stearothermophilus, Bacillus thermocatenulatus, Bacillus thermoleovorans, Bacillus kaustophilus, Bacillus thermodenitrificans* to *Geobacillus* as the new combinations *G. stearothermophilus*, G. th. *Int. J. Syst. Evol. Microbiol.* 51 433–446. 10.1099/00207713-51-2-433 11321089

[B157] Nieminen T. Rintaluoma N. Andersson M. Taimisto A.-M. Ali-Vehmas T. Seppälä A. (2007). Toxinogenic *Bacillus pumilus* and *Bacillus licheniformis* from mastitic milk. *Vet. Microbiol.* 124 329–339. 10.1016/j.vetmic.2007.05.015 17611049

[B158] Nobrega F. L. Costa A. R. Kluskens L. D. Azeredo J. (2015). Revisiting phage therapy: new applications for old resources. *Trends Microbiol.* 23 185–191. 10.1016/j.tim.2015.01.006 25708933

[B159] O’Brien B. Berry D. P. Kelly P. Meaney W. J. O’Callaghan E. J. (2009). *A Study of the Somatic Cell Count (SCC) of Irish Milk from Herd Management and Environmental Perspectives.* Ph.D. thesis, University College Dublin Dublin.

[B160] O’Sullivan D. J. Giblin L. McSweeney P. L. Sheehan J. J. Cotter P. D. (2013). Nucleic acid-based approaches to investigate microbial-related cheese quality defects. *Front. Microbiol.* 4:1 10.3389/fmicb.2013.00001 PMC3549567 23346082

[B161] Paananen A. Mikkola R. Sareneva T. Matikainen S. Hess M. Andersson M. (2002). Inhibition of human natural killer cell activity by cereulide, an emetic toxin from *Bacillus cereus*. *Clin. Exp. Immunol.* 129 420–428. 10.1046/j.1365-2249.2002.01898.x 12197882 PMC1906479

[B162] Palabiyik I. Yilmaz M. T. Fryer P. J. Robbins P. T. Toker O. S. (2015). Minimising the environmental footprint of industrial-scaled cleaning processes by optimisation of a novel clean-in-place system protocol. *J. Clean Prod.* 108 1009–1018. 10.1016/j.jclepro.2015.07.114

[B163] Pantoja J. C. Rosa G. J. Reinemann D. J. Ruegg P. L. (2012). Sampling strategies for total bacterial count of unpasteurized bulk milk. *J*. *Dairy Sci.* 95 2326–2335. 10.3168/jds.2011-5098 22541461

[B164] Parkar S. Flint S. Palmer J. Brooks J. (2001). Factors influencing attachment of thermophilic bacilli to stainless steel. *J*. *Appl. Microbiol.* 90 901–908. 10.1046/j.1365-2672.2001.01323.x 11412320

[B165] Parkar S. G. Flint S. H. Brooks J. D. (2003). Physiology of biofilms of thermophilic bacilli - potential consequences for cleaning. *J. Ind. Microbiol. Biotechnol.* 30 553–560. 10.1007/s10295-003-0081-x 14513382

[B166] Parkar S. G. Flint S. H. Brooks J. D. (2004). Evaluation of the effect of cleaning regimes on biofilms of thermophilic bacilli on stainless steel. *J. Appl. Microbiol.* 96 110–116. 10.1046/j.1365-2672.2003.02136.x 14678164

[B167] Paul T. Jana A. Das A. Mandal A. Halder S. K. Mohapatra P. K. D. (2014). Smart cleaning-in-place process through crude keratinase: an eco-friendly cleaning techniques towards dairy industries. *J. Clean Prod.* 76 140–153. 10.1016/j.jclepro.2014.04.028

[B168] Pavic S. Brett M. Petric I. Lastre D. Smoljanovic M. Atkinson M. (2005). An outbreak of food poisoning in a kindergarten caused by milk powder containing toxigenic *Bacillus subtilis* and *Bacillus licheniformis*. *Arch. Lebensmittel.* 56 20–22.

[B169] Pereira A. Melo L. F. Fratamico P. M. Annous B. A. Gunther N. W. (2009). “Monitoring of biofilms in the food and beverage industries,” in *Biofilms in the Food and Beverage Industries* eds Pereira A. Melo L. F. (Oxford: Wood head Publishing Ltd.) 131–151.

[B170] Pettersson B. Lembke F. Hammer P. Stackebrandt E. Priest F. G. (1996). *Bacillus sporothermodurans*, a new species producing highly heat-resistant endospores. *Int. J. Syst. Bacteriol.* 46 759–764. 10.1099/00207713-46-3-759 8782686

[B171] Phelps R. J. McKillip J. L. (2002). Enterotoxin production in natural isolates of Bacillaceae outside the *Bacillus cereus* group. *Appl. Environ. Microbiol.* 68 3147–3151. 10.1128/AEM.68.6.3147-3151.2002 12039781 PMC123918

[B172] Phillips J. Griffiths M. (1986). Factors contributing to the seasonal variation of *Bacillus* spp. in pasteurized dairy products. *J. Appl. Bacteriol.* 61 275–285. 10.1111/j.1365-2672.1986.tb04288.x 3781939

[B173] Phillips J. D. Griffiths M. W. Nriagu J. Simmons M. (1990). “Pasteurized dairy products: the constraints imposed by environmental contamination,” in *Advances in Environmental Science and Technology* eds Nriagu J. O. Simmons M. S. (New York, NY: John Wiley & Sons, Inc.) 387–454.

[B174] Piyasena P. Mohareb E. McKellar R. C. (2003). Inactivation of microbes using ultrasound: a review. *Int. J. Food Microbiol.* 87 207–216. 10.1016/S0168-1605(03)00075-8 14527793

[B175] Pontefract R. (1991). Bacterial adherence: its consequences in food processing. *Can. Inst. Food Sci. Technol. J.* 24 113–117. 10.1111/j.1750-3841.2010.01936.x

[B176] Postollec F. Mathot A.-G. Bernard M. Divanac’h M.-L. Pavan S. Sohier D. (2012). Tracking spore-forming bacteria in food: from natural biodiversity to selection by processes. *Int. J. Food Microbiol.* 158 1–8. 10.1016/j.ijfoodmicro.2012.03.004 22795797

[B177] Quigley L. McCarthy R. O’Sullivan O. Beresford T. P. Fitzgerald G. F. Ross R. P. (2013a). The microbial content of raw and pasteurized cow milk as determined by molecular approaches. *J. Dairy Sci.* 96 4928–4937. 10.3168/jds.2013-6688 23746589

[B178] Quigley L. O’Sullivan O. Stanton C. Beresford T. P. Ross R. P. Fitzgerad G. F. (2013b). The complex microbiota of raw milk. *FEMS Microbiol. Rev.* 37 664–698. 10.1111/1574-6976.12030 23808865

[B179] Rainey F. A. Fritze D. Stackebrandt E. (1994). The phylogenetic diversity of thermophilic members of the genus *Bacillus* as revealed by 16S rDNA analysis. *FEMS Microbiol. Lett.* 115 205–211. 10.1111/j.1574-6968.1994.tb06639.x 8138135

[B180] Rajkovic A. Uyttendaele M. Vermeulen A. Andjelkovic M. Fitz-James I. in ’t Veld P. (2008). Heat resistance of *Bacillus cereus* emetic toxin, cereulide. *Lett. Appl. Microbiol.* 46 536–541. 10.1111/j.1472-765X.2008.02350.x 18363653

[B181] Ranieri M. Boor K. (2009). Short communication: bacterial ecology of high-temperature, short-time pasteurized milk processed in the United States. *J. Dairy Sci.* 92 4833–4840. 10.3168/jds.2009-2181 19762798

[B182] Ranieri M. Boor K. (2010). Tracking and eliminating sporeformers in dairy systems. *Aust. J. Dairy Technol.* 65 74–80.

[B183] Ranieri M. L. Ivy R. A. Mitchell W. R. Call E. Masiello S. N. Wiedmann M. (2012). Real-time PCR detection of *Paenibacillus* spp. in raw milk to predict shelf life performance of pasteurized fluid milk products. *Appl. Environ. Microbiol.* 78 5855–5863. 10.1128/AEM.01361-12 PMC3406147 22685148

[B184] Raso J. Palop A. Pagan R. Condon S. (1998). Inactivation of *Bacillus subtilis* spores by combining ultrasonic waves under pressure and mild heat treatment. *J. Appl. Microbiol.* 85 849–854. 10.1046/j.1365-2672.1998.00593.x 9830120

[B185] Reddy G. Matsumoto G. Shivaji S. (2003). *Sporosarcina macmurdoensis* sp. nov., from a cyanobacterial mat sample from a pond in the McMurdo Dry Valleys, Antarctica. *Int. J. Syst. Evol. Microbiol.* 53 1363–1367. 10.1099/ijs.0.02628-0 13130019

[B186] Reginensi S. M. Gonzalez M. J. Olivera J. A. Sosa M. Juliano P. Bermudez J. (2011). RAPD-based screening for spore-forming bacterial populations in Uruguayan commercial powdered milk. *Int. J. Food Microbiol.* 148 36–41. 10.1016/j.ijfoodmicro.2011.04.020 21565415

[B187] Roberts T. Hitchins A. (1969). “Resistance of spores,” in *The Bacterial Spore 1* eds Gould G. W. Hurst A. (New York, NY: Academic Press) 611–670.

[B188] Rönner U. Husmark U. Henriksson A. (1990). Adhesion of *Bacillus spores* in relation to hydrophobicity. *J. Appl. Bacteriol.* 69 550–556. 10.1111/j.1365-2672.1990.tb01547.x 2292519

[B189] Rückert A. Ronimus R. S. Morgan H. W. (2004). A RAPD-based survey of thermophilic bacilli in milk powders from different countries. *Int. J. Food Microbiol.* 96 263–272. 10.1016/j.ijfoodmicro.2004.03.020 15454316

[B190] Salkinoja-Salonen M. Vuorio R. Andersson M. Kämpfer P. Andersson M. Honkanen-Buzalski T. (1999). Toxigenic strains of *Bacillus licheniformis* related to food poisoning. *Appl. Environ. Microbiol.* 65 4637–4645. 10508100 10.1128/aem.65.10.4637-4645.1999 PMC91618

[B191] Sanders M. E. Morelli L. Tompkins T. A. (2003). Sporeformers as human probiotics: *Bacillus, Sporolactobacillus* and *Brevibacillus*. *Compr. Rev. Food Sci. Food Saf.* 2 101–110. 10.1111/j.1541-4337.2003.tb00017.x 33451235

[B192] Scheldeman P. Goossens K. Rodriguez-Diaz M. Pil A. Goris J. Herman L. (2004). *Paenibacillus lactis* sp. nov., isolated from raw and heat-treated milk. *Int. J. Syst. Evol. Microbiol.* 54 885–891. 10.1099/ijs.0.02822-0 15143040

[B193] Scheldeman P. Herman L. Foster S. Heyndrickx M. (2006). Bacillus sporothermodurans and other highly heat-resistant spore formers in milk. *J. Appl. Microbiol.* 101 542–555. 10.1111/j.1365-2672.2006.02964.x 16907805

[B194] Scheldeman P. Pil A. Herman L. De Vos P. Heyndrickx M. (2005). Incidence and diversity of potentially highly heat-resistant spores isolated at dairy farms. *Appl. Environ. Microbiol.* 71 1480–1494. 10.1128/AEM.71.3.1480-1494.2005 15746351 PMC1065131

[B195] Schoeni J. L. (2005). *Bacillus cereus* food poisoning and its toxins. *J. Food Protect.* 68 636–648. 10.4315/0362-028x-68.3.636 15771198

[B196] Scott S. A. Brooks J. D. Rakonjac J. Walker K. M. Flint S. H. (2007). The formation of thermophilic spores during the manufacture of whole milk powder. *Int. J. Dairy Technol.* 60 109–117. 10.1111/j.1471-0307.2007.00309.x

[B197] Seale R. Flint S. McQuillan A. Bremer P. (2008). Recovery of spores from thermophilic dairy bacilli and effects of their surface characteristics on attachment to different surfaces. *Appl. Environ. Microbiol.* 74 731–737. 10.1128/AEM.01725-07 18083853 PMC2227720

[B198] Seale R. B. Dhakal R. Chauhan K. Craven H. M. Deeth H. C. Pillidge C. J. (2012). Genotyping of present-day and historical Geobacillus species isolates from milk powders by high-resolution melt analysis of multiple variable-number tandem-repeat loci. *Appl. Environ. Microbiol.* 78 7090–7097. 10.1128/AEM.01817-12 22865061 PMC3457474

[B199] Shaheen R. Svensson B. Andersson M. A. Christiansson A. Salkinoja-Salonen M. (2010). Persistence strategies of *Bacillus cereus* spores isolated from dairy silo tanks. *Food Microbiol.* 27 347–355. 10.1016/j.fm.2009.11.004 20227599

[B200] Sheehan J. D. (2011). “Cheese avoidance of gas blowing,” in *Encyclopedia of Dairy Science* 2nd Edn ed. Fuquay J. W. (San Diego, CA: Academic Press) 661–666.

[B201] Sheehan J. D. (2013). Milk quality and cheese diversification. *Irish J. Agric. Food Res.* 52 243–253.

[B202] Shida O. Takagi H. Kadowaki K. Komagata K. (1996). Proposal for two new genera, *Brevibacillus* gen. nov. and *Aneurinibacillus* gen. nov. *Int. J. Syst. Bacteriol.* 46 939–946. 10.1099/00207713-46-4-939 8863420

[B203] Shinagawa K. Konuma H. Sekita H. Sugii S. (1995). Emesis of rhesus monkeys induced by intragastric administration with the HEp-2 vacuolation factor (cereulide) produced by *Bacillus cereus*. *FEMS Microbiol. Lett.* 130 87–90. 10.1111/j.1574-6968.1995.tb07703.x 7557302

[B204] Sillankorva S. Neubauer P. Azeredo J. (2010). Phage control of dual species biofilms of *Pseudomonas fluorescens* and *Staphylococcus lentus*. *Biofouling* 26 567–575. 10.1080/08927014.2010.494251 20544433

[B205] Sillankorva S. Oliveira R. R. Vieira M.-J. Sutherland I. Azeredo J. (2004). Bacteriophage Φ S1 infection of *Pseudomonas fluorescens* planktonic cells versus biofilms. *Biofouling* 20 133–138. 10.1080/08927010410001723834 15545062

[B206] Simões M. Simões L. C. Vieira M. J. (2010). A review of current and emergent biofilm control strategies. *LWT-Food Sci. Technol.* 43 573–583. 10.1016/j.lwt.2009.12.008

[B207] Slepecky R. A. Hemphill H. E. (2006). The genus *Bacillus*—nonmedical. *Prokaryotes* 4 530–562. 10.1007/0-387-30744-3_16

[B208] Sobrino-López A. Martín-Belloso O. (2008). Use of nisin and other bacteriocins for preservation of dairy products. *Int. Dairy J.* 18 329–343. 10.1016/j.idairyj.2007.11.009

[B209] Somers J. M. Kelly A. L. (2002). Contribution of plasmin to primary proteolysis during ripening of cheese: effect of milk heat treatment and cheese cooking temperature. *Le Lait* 82 181–191. 10.1051/lait:2002003

[B210] Srey S. Jahid I. K. Ha S.-D. (2013). Biofilm formation in food industries: a food safety concern. *Food Control* 31 572–585. 10.1016/j.foodcont.2012.12.001

[B211] Stark P. L. Lee A. (1982). The microbial ecology of the large bowel of breastfed and formula-fed infants during the first year of life. *J. Med. Microbiol.* 15 189–203. 10.1099/00222615-15-2-189 7143428

[B212] Stenfors Arnesen L. Granum P. E. Buisson C. Bohlin J. Nielsen-LeRoux C. (2011). Using an insect model to assess correlation between temperature and virulence in *Bacillus weihenstephanensis* and *Bacillus cereus*. *FEMS Microbiol. Lett.* 317 196–202. 10.1111/j.1574-6968.2011.02229.x 21276046

[B213] Stenfors Arnesen L. P. Fagerlund A. Granum P. E. (2008). From soil to gut: *Bacillus cereus* and its food poisoning toxins. *FEMS Microbiol. Rev.* 32 579–606. 10.1111/j.1574-6976.2008.00112.x 18422617

[B214] Storgards E. Simola H. Sjöberg A.-M. Wirtanen G. (1999a). Hygiene of gasket materials used in food processing equipment part 1: new materials. *Food Bioprod. Process* 77 137–145.

[B215] Storgards E. Simola H. Sjöberg A.-M. Wirtanen G. (1999b). Hygiene of gasket materials used in food processing equipment part 2: aged materials. *Food Bioprod. Process* 77 146–155. 10.1205/096030899532295

[B216] Stratton J. E. Hutkins R. W. Taylor S. L. (1991). Biogenic amines in cheese and other fermented foods: a review. *J. Food Protect.* 6 408–475. 10.4315/0362-028X-54.6.460 31051616

[B217] Sung M. Kim H. Bae J. Rhee S. Jeon C. Kim K. (2002). *Geobacillus toebii* sp. nov., a novel thermophilic bacterium isolated from hay compost. *Int. J. Syst. Evol. Microbiol.* 52 2251–2255. 10.1099/00207713-52-6-2251 12508894

[B218] Suominen I. Andersson M. A. Andersson M. C. Hallaksela A.-M. Kämpfer P. Rainey F. A. (2001). Toxic *Bacillus pumilus* from indoor air, recycled paper pulp, Norway spruce, food poisoning outbreaks and clinical samples. *Syst. Appl. Microbiol.* 24 267–276. 10.1078/0723-2020-00025 11518331

[B219] Sutherland A. D. Murdoch R. (1994). Seasonal occurrence of psychrotrophic *Bacillus species* in raw milk, and studies on the interactions with mesophilic *Bacillus* sp. *Int. J. Food Microbiol.* 21 279–292. 10.1016/0168-1605(94)90058-2 8043347

[B220] Tachikawa M. Yamanaka K. (2014). Synergistic disinfection and removal of biofilms by a sequential two-step treatment with ozone followed by hydrogen peroxide. *Water Res.* 64 94–101. 10.1016/j.watres.2014.06.047 25043797

[B221] Tait K. Skillman L. C. Sutherland I. W. (2002). The efficacy of bacteriophage as a method of biofilm eradication. *Biofouling* 18 305–311. 10.1111/j.1365-2672.2008.03743.x

[B222] Tamime A. Y. (2008). “Cleaning-in-Place,” in *Dairy, Food and Beverages Operations* 3rd Edn ed. Tamime A. Y. (Oxford: Blackwell Publishing).

[B223] Tan S. Y.-E. Chew S. C. Tan S. Y.-Y. Givskov M. Yang L. (2014). Emerging frontiers in detection and control of bacterial biofilms. *Curr. Opin. Biotechnol.* 26 1–6. 10.1016/j.copbio.2013.08.002 24679251

[B224] Tatzel R. Ludwig W. Schleifer K. H. Wallnfer P. R. (1994). Identification of *Bacillus* strains isolated from milk and cream with classical and nucleic acid hybridization methods. *J. Dairy Res.* 61 529–535. 10.1017/S0022029900028454 7829756

[B225] Taylor J. M. Sutherland A. D. Aidoo K. E. Logan N. A. (2005). Heat-stable toxin production by strains of *Bacillus cereus, Bacillus firmus, Bacillus megaterium, Bacillus* simplex and *Bacillus licheniformis*. *FEMS Microbiol. Lett.* 242 313–317. 10.1016/j.femsle.2004.11.022 15621453

[B226] te Giffel M. T. Wagendorp A. Herrewegh A. Driehuis F. (2002). Bacterial spores in silage and raw milk. *Antonie Van Leeuwenhoek* 81 625–630. 10.1023/A:1020578110353 12448758

[B227] Teh K. H. Flint S. Palmer J. Andrewes P. Bremer P. Lindsay D. (2014). Biofilm- An unrecognised source of spoilage enzymes in dairy products? *Int. Dairy J.* 34 32–40. 10.1016/j.idairyj.2013.07.002

[B228] Teixeira P. Rodrigues D. (2014). “Antibiofilm strategies in the food industry,” in *Antibiofilm Agents Springer Series on Biofilms* eds Rumbaugh K. P. Ahmad I. (Berlin: Springer-Verlag) 359–381. 10.1007/978-3-642-53833-9_16

[B229] Thorsen L. Hansen B. M. Nielsen K. F. Hendriksen N. B. Phipps R. K. Budde B. B. (2006). Characterization of emetic *Bacillus weihenstephanensis*, a new cereulide-producing bacterium. *Appl. Environ. Microbiol.* 72 5118–5121. 10.1128/AEM.00170-06 16820519 PMC1489381

[B230] Tolle A. (1980). “The microflora of the udder,” in *Factors Influencing the Bacteriological Quality Of Raw Milk, International Dairy Federation Bulletin, Document 120* 4.

[B231] Tominaga T. An S.-Y. Oyaizu H. Yokota A. (2009). *Sporosarcina luteola* sp. nov. isolated from soy sauce production equipment in Japan. *J. Gen. Appl. Microbiol.* 55 217–223. 10.2323/jgam.55.217 19590149

[B232] Turnbull P. C. Jackson P. J. Hill K. K. Keim P. Kolstø A. B. Beecher D. J. (2002). “Longstanding taxonomic enigmas within the ‘*Bacillus cereus* group’ are on the verge of being resolved by far-reaching molecular developments: forecasts on the possible outcome by an ad hoc team,” in *Applications and Systematics of Bacillus and Relatives* eds Berkeley R. Heyndrickx M. Logan N. De Vos P. (Oxford: Blackwell Publishing) 23–36.

[B233] Vaerewijck M. De Vos P. Lebbe L. Scheldeman P. Hoste B. Heyndrickx M. (2001). Occurrence of *Bacillus sporothermodurans* and other aerobic spore-forming species in feed concentrate for dairy cattle. *J. Appl. Microbiol.* 91 1074–1084. 10.1046/j.1365-2672.2001.01477.x 11851816

[B234] Vandamme E. J. Miedzybrodzki R. (2013). Phage therapy and phage control: to be revisited urgently! *J. Chem. Technol. Biotechnol.* 89 329–333. 10.1002/jctb.4245

[B235] Van Houdt R. Michiels C. W. (2010). Biofilm formation and the food industry, a focus on the bacterial outer surface. *J. Appl. Microbiol.* 109 1117–1131. 10.1111/j.1365-2672.2010.04756.x 20522145

[B236] Vedamuthu E. (1992). The yogurt story: past, present and future. III. *Dairy Food Environ. Sanit.* 11 310–311.

[B237] Villamiel M. Schutyserm M. A. I. De Jong P. (2009). “8. novel methods of milk processing,” in *Milk Processing and Quality Management* ed. Tamime A. Y. (Hoboken, NJ Blackwell Publishing Ltd.)

[B238] Vlková H. Babák V. Seydlová R. Pavlík I. Schlegelová J. (2008). Biofilms and hygiene on dairy farms and in the dairy industry: sanitation chemical products and their effectiveness on biofilms- a review. *Czech J. Food Sci.* 26 309–323.

[B239] Walsh D. Molloy C. Iversen C. Carroll J. Cagney C. Fanning S. (2011). Survival characteristics of environmental and clinically derived strains of *Cronobacter sakazakii* in infant milk formula (IMF) and ingredients. *J. Appl. Microbiol.* 110 697–703. 10.1111/j.1365-2672.2010.04921.x 21255207

[B240] Walton M. (2008). “1. Principles of cleaning-in-place (CIP),” in *Cleaning-In-Place: Dairy, Food and Beverage Operations* 3rd Edn ed. Tamime A. Y. (Hoboken, NJ: Blackwell Publishing Ltd.).

[B241] Watterson M. J. Kent D. J. Boor K. J. Wiedmann M. Martin N. H. (2014). Evaluation of dairy powder products implicates thermophilic sporeformers as the primary organisms of interest. *J. Dairy Sci.* 97 2487–2497. 10.3168/jds.2013-7363 24485694

[B242] White J. C. Rabe G. O. (1970). Evaluating the use of nitric acid as a detergent in dairy cleaned-in-place systems. *J. Milk Food Technol.* 33 25–28.

[B243] Wiencek K. M. Klapes N. A. Foegeding P. M. (1991). Adhesion of *Bacillus* spores to inanimate materials: effects of substratum and spore hydrophobicity. *Biofouling* 3 139–149. 10.1080/08927019109378168

[B244] Wolfgang W. J. Coorevits A. Cole J. A. De Vos P. Dickinson M. C. Hannett G. E. (2012). *Sporosarcina newyorkensis* sp. nov. from clinical specimens and raw cow’s milk. *Int. J. Syst. Evol. Microbiol.* 62 322–329. 10.1099/ijs.0.030080-0 21421928

[B245] Xu D. Côté J. C. (2003). Phylogenetic relationships between *Bacillus* species and related genera inferred from comparison of 3′ end 16S rDNA and 5′ end 16S-23S ITS nucleotide sequences. *Int. J. Syst. Evol. Microbiol.* 53(Pt 3) 695–704. 10.1099/ijs.0.02346-0 12807189

[B246] Xu J. Bigelow T. A. Halverson L. J. Middendorf J. M. Rusk B. (2012). Minimization of treatment time for in vitro 1.1 MHz destruction of *Pseudomonas aeruginosa* biofilms by high-intensity focused ultrasound. *Ultrasonics* 52 668–675. 10.1016/j.ultras.2012.01.013 22341761

[B247] Yoo J.-A. Hardin M. T. Chen X. D. (2006). The influence of milk composition on the growth of *Bacillus stearothermophilus*. *J. Food Eng.* 77 96–102. 10.1016/j.jfoodeng.2005.06.053

[B248] Yoo J. G. Chang J.-H. Kim S.-Y. Ji J.-Y. Hong S.-W. Park B.-Y. (2014). Analysis of emetic toxin production by *Bacillus species* using cellular cytotoxicity, molecular, and chromatographic assays. *Biotechnol. Bioprocess. Eng.* 19 978–983. 10.1007/s12257-014-0574-7

[B249] Yoon J.-H. Lee K.-C. Weiss N. Kho Y. H. Kang K. H. Park Y.-H. (2001). *Sporosarcina aquimarina* sp. nov., a bacterium isolated from seawater in Korea, and transfer of. *Bacillus globisporus* (Larkin and Stokes 1967), *Bacillus psychrophilus* (Nakamura 1984) and *Bacillus pasteurii* (Chester 1898) to the genus *Sporosarcina* as *Sporosarcina globispora* comb. nov., *Sporosarcina psychrophila* comb. nov. and *Sporosarcina pasteurii* comb. nov., and emended description of th. *Int. J. Syst. Evol. Microbiol.* 51 1079–1086. 10.1099/00207713-51-3-1079 11411676

[B250] Yu Y. Xin Y.-H. Liu H.-C. Chen B. Sheng J. Chi Z.-M. (2008). *Sporosarcina antarctica* sp. nov., a psychrophilic bacterium isolated from the Antarctic. *Int. J. Syst. Evol. Microbiol.* 58 2114–2117. 10.1099/ijs.0.65838-0 18768614

[B251] Yuan D.-D. Liu G.-C. Ren D.-Y. Zhang D. Zhao L. Kan C.-P. (2012). A survey on occurrence of thermophilic bacilli in commercial milk powders in China. *Food Control* 25 752–757. 10.1016/j.foodcont.2011.12.020

[B252] Zaman M. Z. Bakar F. A. SelaMat J. Bakar J. (2010). Occurrence of biogenic amines and amines degrading bacteria in fish sauce. *Czech J. Food Sci.* 28 440–449.

[B253] Zhang W. Lu Z. (2015). Phylogenomic evaluation of members above the species level within the phylum Firmicutes based on conserved proteins. *Environ. Microbiol. Rep.* 7 273–281. 10.1111/1758-2229.12241 25403554

[B254] Zhao Y. Caspers M. P. Metselaar K. I. de Boer P. Roeselers G. Moezelaar R. (2013). Abiotic and microbiotic factors controlling biofilm formation by thermophilic sporeformers. *Appl. Environ. Microbiol.* 79 5652–5660. 10.1128/AEM.00949-13 23851093 PMC3754176

